# Molecular docking, synthesis, kinetics study, structure–activity relationship and ADMET analysis of morin analogous as *Helicobacter pylori* urease inhibitors

**DOI:** 10.1186/s13065-019-0562-2

**Published:** 2019-04-01

**Authors:** Ritu Kataria, Anurag Khatkar

**Affiliations:** 1 International Institute of Pharmaceutical Sciences, Sonepat, Haryana India; 2 Laboratory for Preservation Technology and Enzyme Inhibition Studies, Department of Pharmaceutical Sciences, Faculty of Pharmaceutical Sciences, Maharshi Dayanand University, Rohtak, Haryana India

**Keywords:** Morin, Urease inhibition, Natural phenolic compounds, Antioxidant

## Abstract

**Background:**

Urease are responsible for several pathogenic states in human as well as in animals and its inhibition is utmost urgent. Clinically used drugs are associated with many side effects; recently several researches have shown that flavonoids have good urease inhibition properties. Morin, a natural flavonoid has been investigated for urease inhibition studies which includes designing of library of morin analogues and their in-silico evaluation with the help of Schrodinger’s maestro package of molecular docking software against crystallographic complex of plant enzyme Jack bean urease (PDB ID: 3LA4) followed by synthesis and in vitro evaluation.

**Results:**

Best thirteen derivatives of morin were selected on the basis of their interaction energy and dock score for synthesis and further investigated for in-vitro antioxidant, urease inhibitory and *Anti*-*H. Pylori* activity. In-vitro results revealed that a large number of synthesized compounds were found to possess excellent antioxidant and urease Inhibition properties.

**Conclusions:**

Among the synthesized compounds, *N*-*(2*-*chlorophenyl)*-*N*-*((4E)*-*2*-*(2,4*-*dihydroxyphenyl)*-*3,5,7*-*trihydroxy*-*4H*-*chromen*-*4*-*ylidene)thiourea* (M2b) and *N*-*(4*-*bromophenyl)*-*N*-*((4E)*-*2*-*(2,4*-*dihydroxyphenyl)*-*3,5,7*-*trihydroxy*-*4H*-*chromen*-*4*-*ylidene)thiourea* (M2i) were found to be most potent urease inhibitor and antioxidant with IC_50_ value 10.74 ± 0.018, 11.12 ± 0.033 and 7.37 ± 0.024, 7.73 ± 0.015and 7.795 ± 0.003 µM. Derivative M2i exhibited good anti-*H. pylori* activity having MIC = 500 *μ*g/ml and zone of inhibition 15.00 ± 0.00 mm as compared to standard AHA having MIC = 1000 *μ*g/ml and zone of inhibition 9.00 ± 0.50 mm determined against *H. Pylori* bacterium (ATCC 43504, DSM 4867) by well diffusion technique. Furthermore, molecular docking study explained the binding pattern of synthesized ligand within active cavity of jack bean protein and drug similarity was explained by ADME studies by quikprop module of molecular docking software.

**Electronic supplementary material:**

The online version of this article (10.1186/s13065-019-0562-2) contains supplementary material, which is available to authorized users.

## Introduction

Urease (urea amidohydrolase; E.C. 3.5.1.5) is a nickel containing metalloenzyme brings catalytic hydrolysis of urea and leads to the formation of ammonia and carbamate which instinctively disintegrates, at normal functioning pH, to give another ammonia molecule and bicarbonate [1]. It’s presence in soil was first reported by Rotini [2]. It increases rate of biochemical dissociation of urea by 10^14^ times [3]. Urease plays role of key enzyme for global nitrogen cycle and supplies nitrogen to plants for seed germination and for growth [4]. However, high urease action is responsible for release of unusually a lot of ammonia into climate which may prompt natural and monetary issues [1, 4]. Ureases have been found among plants, microscopic organisms like bacteria, algae, fungi and invertebrates [12]. Catalytic mechanism of plant and microbial originated urease is similar although they possesses structural differences, probably as they have similar pattern of amino acids and Ni^+2^ ions at active center which indicates its emergence from a common ancestry [4–6].

Urease are responsible for several pathogenic states in both human as well as in animals such as urinary and GIT infections, gastric cancer, stone formation in kidney, pyelonephritis, encrustation in catheter, ammonia encephalopathy, hepatic coma [7–11]. Urease is also a virulence factor found in pathogenic bacteria *H. Pylori* and is one of the main cause for its spreading in gastric environment by catalyzing the urea present there. Released ammonia thereby causes elevation in pH and makes a comfortable environment for pathogen *H. Pylori* to survive and spread colonies. The presence of excess of urease cause breakdown of fertilizer urea and ammonia released in high concentration in the climate and in addition plants get damaged due to toxicity of ammonia and elevation in soil pH, consequently posturing noteworthy ecological and financial troubles [10].

Morin (3, 5, 7, 2′, 4′-pentahydroxyflavone), a bioflavonoid found in fruits, vegetables and various medicinal herbs such as figs, mulberry, Osage orange (*Maclura pomifera*), sweet chestnut (*Castanea sativa*, Family-*Fagaceae*) guava (*Psidium guajana* L.) leaves, Brazilian wood (*Chlorophora tinctoria*), almond (*Prunus dulcis*, Family-*Rosaceae*), onion, seaweeds, apple, Chinese and Asian medicinal herb as well as in several beverages [12–18] was initially isolated from the *Moraceae* family [12]. It has been documented that morin is enriched with wide range of biological activities like anti-inflammatory activity [13], antiallergen [15], hepatoprotective [19], antihypertensive, antioxidant, nephroprotective, antidiabetic, anticlastogenic activities, cytoprotective effects, xanthine oxidase–hypoxanthine activity decreases the frequency of septic shock [14] and various types of cancers like liver cancer, breast cancer, colon cancer, oral tumor and have been used as an herbal medicine from long time [10–21].

**Fig. 1:**
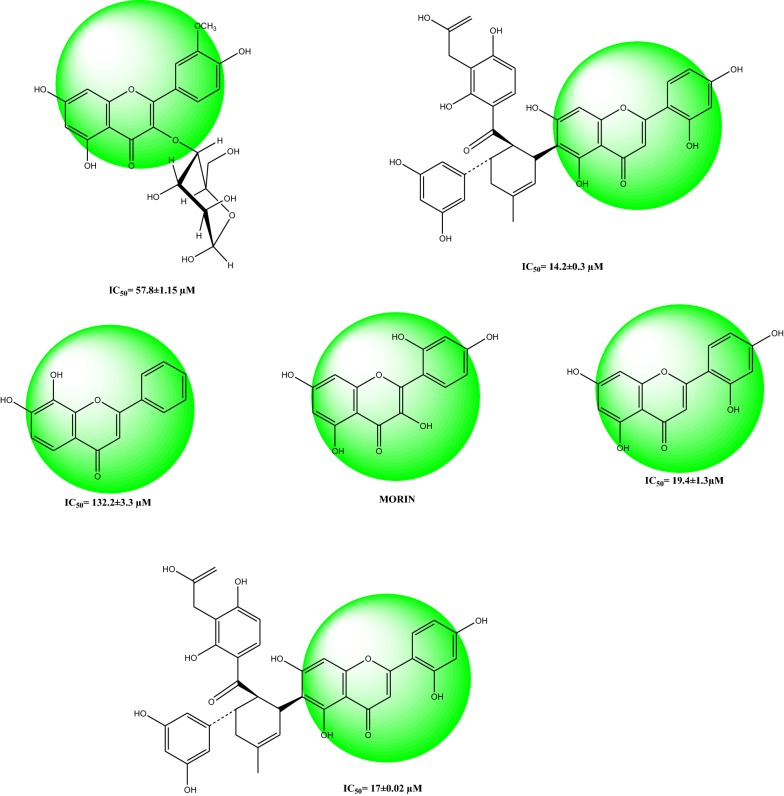
Reported flavonoids having same pharmacophore with morin possessing urease inhibitory activity. flavonoids

Drugs used clinically for the treatment of ailments caused by urease producing bacteria includes bismuth complex, phosphoramidates, imidazole derivatives and hydroxamic acids are associated with numerous side effects like teratogenic effects shown by hydroxamic acid and rapid disintegration of phosphoramidates at low pH. Pharmaceutical industry is in continuous search for better safe and effective drug for treating such disease. Naturally occurring flavonoids have shown urease inhibitory as well as anti *H. Pylori* activity as reported by several researchers. Awllia Jalaluddin et al. [22] recently evaluated nine flavonoids for inhibition of jack bean urease and five of them were found to be good in activity having IC_50_ values 14.2 ± 0.3 to 132.9 ± 3.3 *μ*M shown in Fig. 1 [23]. Similarity in pharmacophore of morin with these flavonoids i.e. 2-phenyl-4H-chromen-4-one and pharmacological enrichment of morin motivated the researchers to design more potent derivatives of it by using docking studies and then evaluation was done for antioxidant, urease inhibition and anti-*H. Pylori* activity by in-vitro studies.

## Results and discussion

### Chemistry

**Scheme 1:**
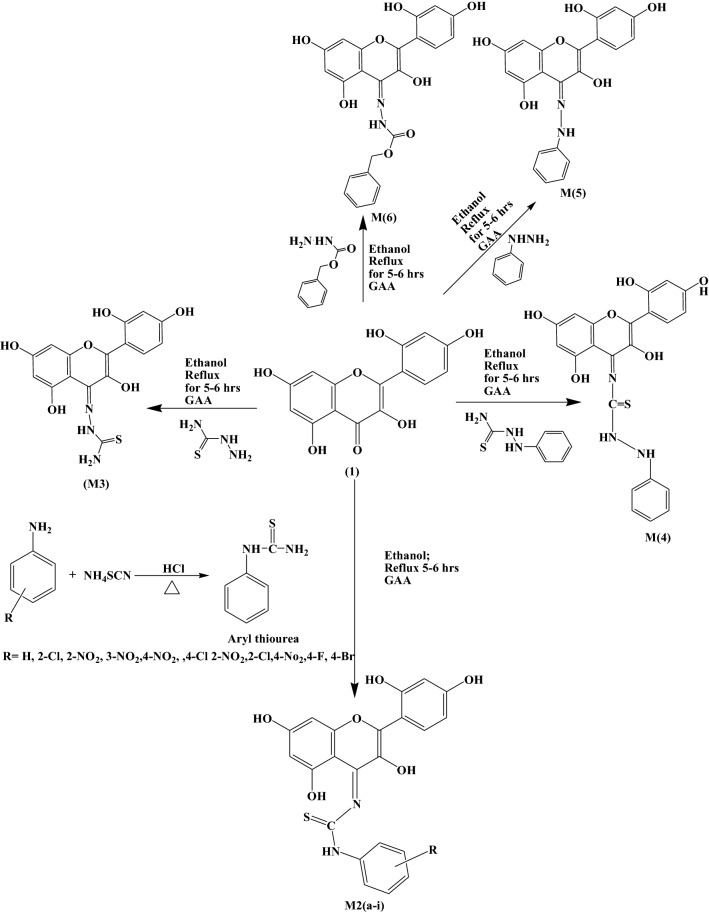
Synthetic scheme for the synthesis of morin derivatives

In the current study, morin derivatives **(M2a–i, M3–6)** were synthesized by following the procedure illustrated in Scheme 1 [24–26]. Synthetic scheme involve two-step process involving formation of substituted aryl thiourea by reaction of substituted anilines with ammonium thiocyanate in presence of hydrochloric acid. Morin derivatives were synthesized in second step by reaction with equimolar concentration of arylthiourea(M2a-i)/thiosemicarbazide(M3)/phenylthiosemicarbazide(M4)/phenyhydrazine(M5)/benzylcarbazate(M6) in ethanol with glacial acetic acid as catalyst. Monitoring of reaction was done by thin layer chromatography and completion of reaction was confirmed by single spot in TLC under UV lamp. Evaluation of structure of novel derivatives was confirmed by spectroscopic methods such as IR, ^1^H NMR, ^13^CNMR, elemental analysis. Derivatisation was confirmed by downward shift of peak at 1680 (C=O)str to 1615–1625 (C=N)str. Appearance of a peak at 1539–1571 of N–C=S str was observed in compound M2a–M2i which further confirmed the formation of derivatives. Whereas in compounds M3, M4, M5 and M6 additional peak at 1033–1078 for N–Nstr was also observed which confirmed formation of M3–M6 compounds. ^1^HNMR signals were interpreted by their value of chemical shift for particular protons of synthesized derivatives, coupling constant and multiplicities of signals. For instance in compound M2a appearance of singlet at δ 12.68, 10.68, 9.7, 9.68 was noticed for OH, OH, NH and OH groups respectively and 7.53–6.30 for aromatic protons, wheras in ^13^C NMR signals at δ 187 (C–S) and 157 (C–N) confirmed formation of compound. Further and final confirmation process involves analyzing their mass spectrum for determination of molecular weight in which Q-ToF Micro instrument was used as ion source. Maximum number of the derivatives showed peak at M^+^ (molecular ion peak), (M^++1^), (M^++2^) in positive chemical ionization and (M^+1^), (M^+2^), M^+^ during negative chemical ionization mode. Elemental analysis of diosmin derivative was carried out in CHNS analyzer where C, H and N in percent were found within acceptable range.

### Biological activity

**Fig. 2:**
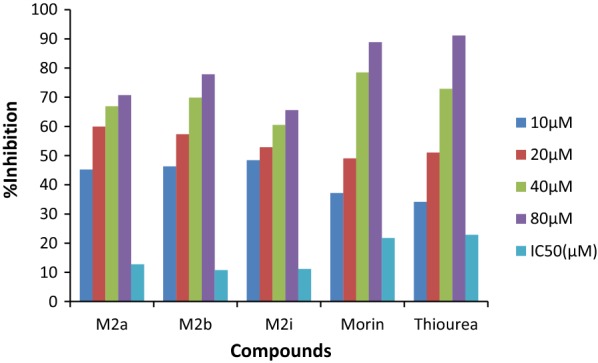
In-vitro urease inhibitory activity of most potent compounds

**Fig. 3:**
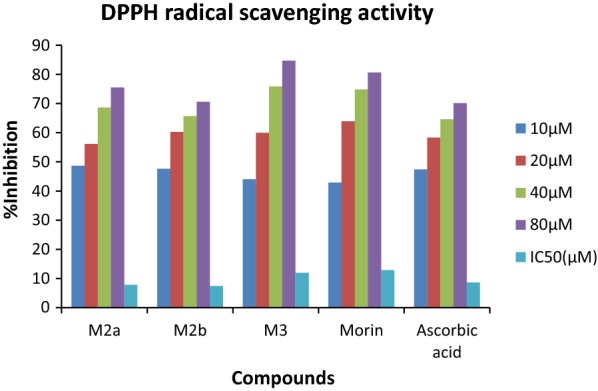
DPPH radical scavenging activity of most potent compounds

**Fig. 4:**
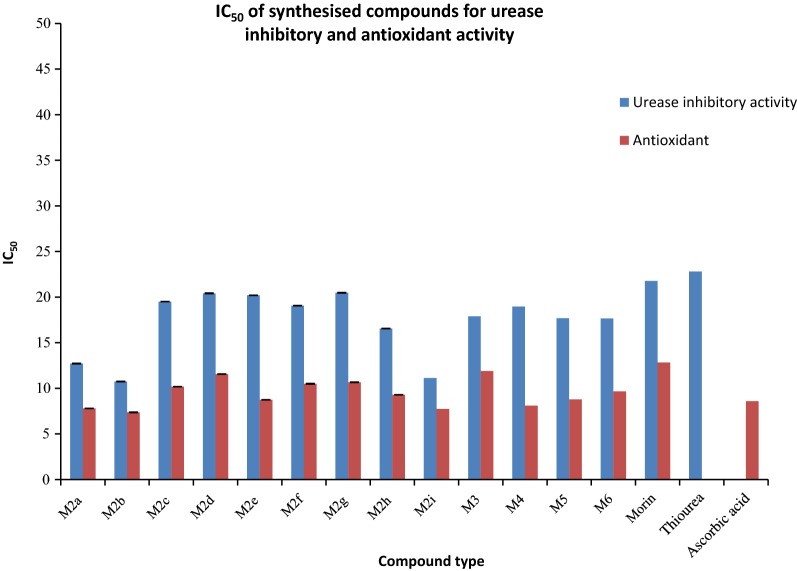
IC_50_ value synthesized compounds for urease inhibition and antioxidant activity

The novel synthesized Morin derivatives were examined for *in*- *vitro* urease inhibitory action by determining the ammonia concentration released during the reaction by indophenol technique as described by Weatherburn [27] as well as for antioxidant nature [28, 29] by determining the ability of compounds to donate hydrogen and electrons using DPPH method. All the synthesized compounds were found to be potent inhibitors of jack bean urease and showed good antioxidant potential with IC_50_ value ranging between 10.74–20.48 µM and 7.37–11.9 µM respectively. Among them compounds M2b, M2i and M2a displayed the excellent urease inhibition with IC_50_ value 10.74 ± 0.018, 11.12 ± 0.033 and 12.71 ± 0.027 µM even two folds more active than standard drug thiourea and also displayed good antioxidant behavior with IC_50_ value 7.37 ± 0.024, 7.73 ± 0.015and 7.795 ± 0.003 µM against DPPH using ascorbic acid as standard as shown in Tables 1 and 2 and Figs. 2, 3 and 4.

**Table 1:** In-vitro urease inhibition activity and IC_50_ of synthesized morin derivatives

Compound	IC_50_(µM)^a^	Compound	IC_50_(µM)^a^
M2a	12.71 ± 0.027	**M2i**	11.12 ± 0.033
M2b	10.74 ± 0.018	**M3**	17.9 ± 0.007
M2c	19.5 ± 0.005	**M4**	18.96 ± 0.011
M2d	20.41 ± 0.031	**M5**	17.68 ± 0.08
M2e	20.2 ± 0.01	**M6**	17.66 ± 0.02
M2f	19.06 ± 0.015	**Morin**	21.77 ± 0.016
M2g	20.48 ± 0.026	**Thiourea**	22.80 ± 0.011
M2h	16.55 ± 0.011		

^a^Values related for the evaluated compound absorption which provide 50% inhibition of Urease inhibition action, and are the mean SEM; statistical significance: p < 0.05 against the equivalent IC_50_ values achieved against urease, as identified through ANOVA/Dunnett’s test

**Table 2:** In-vitro DPPH radical scavenging activities and IC_50_ of synthesized morin derivatives (antioxidant activity)

Compound	IC_50_ (µM)^a^	Compound	IC_50_ (µM)^a^
M2a	7.795 ± 0.003	**M2i**	7.73 ± 0.015
M2b	7.37 ± 0.024	**M3**	11.9 ± 0.031
M2c	10.18 ± 0.006	**M4**	8.099 ± 0.018
M2d	11.56 ± 0.013	**M5**	8.78 ± 0.008
M2e	8.744 ± 0.008	**M6**	9.65 ± 0.022
M2f	10.50 ± 0.036	**Morin**	12.83 ± 0.028
M2g	10.66 ± 0.019	**Ascorbic acid**	8.59 ± 0.004
M2h	9.290 ± 0.007		

^a^Values related for the evaluated compound absorption which provide 50% inhibition of Urease inhibition action, and are the mean SEM; statistical significance: p < 0.05 against the equivalent IC_50_ values achieved against urease, as identified through ANOVA/Dunnett’s test

Compounds **M2b** and **M2i** which were found potent in urease inhibition and antioxidant activity as well as in terms of docking score were tested further against antibacterial efficiency against *Helicobacter pylori* ATCC 43504, DSM 4867 using AHA as a standard and DMSO as control. MIC_50_ values were calculated for compounds and the results revealed that **M2i** displayed its potency with good zone of inhibition i.e. 15.00 ± 0.00 mm as compared to standard 9.00 ± 0.50 mm and MIC_50_ value was found to be comparable to Standard i.e. 500 µg/ml. It could be concluded that compound **M2i** indicated excellent antibacterial action against *H. Pylori*, antioxidant and urease inhibitory activity.

### Enzyme kinetics

**Fig. 5:**
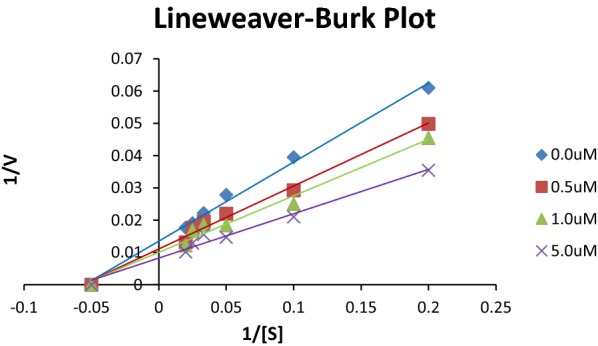
Lineweaver-burke plot for compound M2i

Study of inhibitory effect of morin derivatives on jack bean urease was performed to check the inhibitory potential, kinetics studies and mechanism of inhibition in phosphate buffer and 1 mM EDTA at pH 8.2. Lineweaver–Burk plots (1/absorbance versus 1/urea) was constructed from kinetic data to determine the mechanism of enzyme inhibition by varying the concentration of substrate urea in the presence of different concentrations of most potent compound M2i. Inhibition constant (Ki) was determined as the intersection on x-axis of the plot of 1/Vmax and varying concentration of inhibitor obtained from Lineweaver-Burke plot and all experiments were performed in triplicate. Inhibition was found to be non-competitive as Km was constant but Vmax was changed. Binding confirmations from molecular simulation studies also confirm the mode of inhibition as shown in Fig. 5.

### Structure activity relationship

**Fig. 6:**
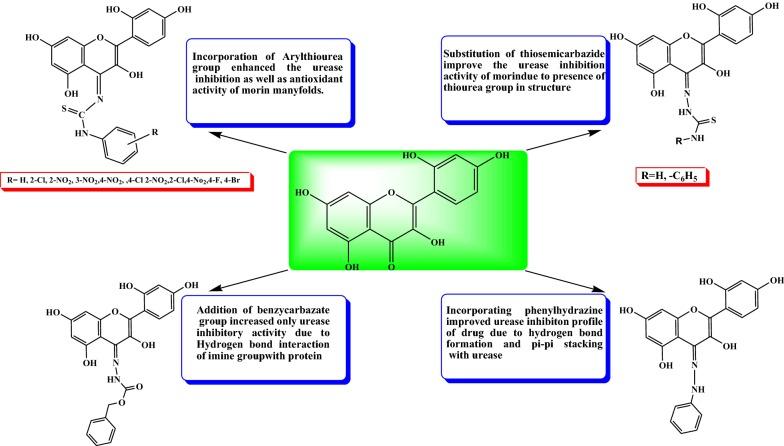
Structure activity relationship of synthesized derivative of morin

Studying results from antioxidant nature and urease inhibition activities of newly synthesized morin derivatives, structure activity relationship can be derived (Fig. 6).

1. Incorporation of substituted arylthiourea group enhanced the urease inhibition as well as antioxidant behavior of morin manyfolds such as compounds (M2a–M2i) having IC_50_ value for urease inhibition from 10.74 ± 0.018 to 20.48 ± 0.026 µM.
2. Attachment of bromo substituted arylthiourea increased the effectiveness of molecule (M2i) in inhibiting growth of pathogenic bacteria *H. Pylori* with MIC value 500 µg/ml and zone of inhibition 15.00 ± 0.00 mm as compared to standard acetohydroxamic acid having MIC 500 µg/ml and zone of inhibition 9.00 ± 0.50 mm.
3. Substitution of thiosemicarbazide and phenylthiosemicarbazide groups in morin leads to improvement of urease inhibition activity to greater extent due to presence of thiourea groups in the structure.
4. Phenylhydrazine and benzylcarbazate addition improved of activity of morin due to stronger interaction of compounds with enzyme through H-bond formation by imine group and pi–pi stacking interactions.
5. Presence of hydroxyl groups improved the antioxidant activity.

### Molecular docking study

Newly designed ligands were studied for molecular simulation studies with the help of Schrodinger’s maestro package of molecular docking software [30–34]. Molecules were docked into Jack bean urease crystallographic complex (PDB ID: 3LA4) by Induced fit docking (IFD) method. The predicted binding pattern revealed that synthesized ligand bind within catalytic cavity firmly via. hydrogen bond formation, pi–pi stacking and hydrophobic interaction. Position and alignment of particular substituents on molecules was found to be responsible for perfect binding of ligand with enzyme. In the binding model of the most active compound (**M2b**) eight hydrogen bonds were noticed between residues Lys 709, Glu 718, Try 32, Val 744, Pro 717, Ash 730, Ser 421 with hydroxyl and NH groups of synthesized ligands. Hydrophobic interactions among ligand and residues Ala 16, Leu 13, Leu 839, Pro 717, Phe 712, Tyr 32, Val 744, Met 746, Val 36, Ala37 was noticed. The second most potent compound (**M2i**) was found to form six hydrogen bonds among Pro 717, Ser 421, Glu 742, Try 32, Ash 730 Lys 709 with hydroxyl and NH groups, a salt bridge formation was also observed between oxygen and Lys 716 residue. Ligand was found to interact with Phe 712, Pro 717, Tyr 32, Met 746, Val 744, Pro 743, Val 36, Ala 37, Leu 13, Ala 16, Leu 839 residues hydrophobically. Similarly six hydrogen bond formations was observed in inhibitor of third rank (**M2a**) between Glu 718, Ash 730, Lys 716, Val 744, Met 746 residues and hydroxyl, thiol and NH group of ligand. Docking studies revealed that hydrogen bond interactions fix the ligands firmly and tightly in the active site. In-silico studies and in-vitro results comply with each other, according to the which, compounds **M2b, M2i** and **M2a** were found to be most potent ligands. Molecular docking parameters were shown in Table 3 and interaction pattern of ligands within the pocket were described in Table 4.

**Table 3:** Docking parameters of the designed derivatives

Compound	Docking score	Binding energy	Glide hbond	Glide ecoul	Glide evdw
M2a	− 9.225	− 61.834	− 2.212	− 15.894	− 45.94
M2b	− 10.977	− 59.062	− 2.364	− 13.986	− 45.076
M2c	− 7.94	− 52.778	− 1.989	− 4.563	− 48.214
M2d	− 7.117	− 48.622	− 1.073	− 4.498	− 44.124
M2e	− 8.306	− 52.965	− 1.907	− 4.693	− 48.002
M2f	− 7.634	− 52.788	− 1.34	− 5.445	− 47.343
M2g	− 7.202	− 55.996	− 0.83	− 7.193	− 48.803
M2h	− 8.576	− 48.949	− 1.65	− 4.621	− 44.329
M2i	− 10.273	− 45.27	− 2.955	− 11.623	− 33.6447
M3	− 8.266	− 47.885	− 1.293	− 12.371	− 35.514
M4	− 8.12	− 54.757	− 1.81	− 10.458	− 44.298
M5	− 8.306	− 49.449	− 1.69	− 7.449	− 42
M6	− 8.536	− 59.562	− 1.424	− 12.371	− 47.323
Thiourea	− 3.459	− 21.156	− 1.484	− 8.152	− 13.004
AHA	− 3.049	− 17.454	− 1.311	− 8.523	− 8.936

**Table 4:** Protein-ligand interaction details of synthesized derivatives

Ligands	Interaction diagram	Interaction description
**M2a**		Hydrogen bond interaction: Glu 718, Ash 730, Lys 716, Val 744, Met 746 Hydrophobic interaction: Phe 712, Pro 717, Try 32, Val 36, Ala 37, Ala 16, Leu 13, Pro 743, Val 744, Met 746, Leu 839
**M2b**		Hydrogen bond interaction: Lys 709, Glu 718, Tyr 32, Val 744, Pro 717, Ash 730, Ser 421 Hydrophobic interaction: Pro 717, Phe 712, Tyr 32, Val 744, Val 36, Met 746, Ala 37, Ala 16, Leu 13, Leu 839
**M2c**		Hydrogen bond interaction: Phe 712, Val 744, Lys 716, Leu 839 Hydrophobic interaction: Phe 712, Phe 840, Leu 839, Phe 838, Tyr 837, Val 744, Pro 743, Ala 16, Leu 13, Ala 37, Val, 36, Tyr 32
**M2d**		Hydrogen bond interaction: Ash 730, Glu 742 Hydrophobic interaction: Phe 712, Pro 717, Tyr 32, Val 36, Ala 37, Pro 743, Val 744, Met 746, Leu 839, Phe 838, Leu 13, Ala 16
**M2e**		Hydrogen bond interaction: Val 744, Glu 742 Hydrophobic interaction: Trp 728, Phe 712, Met 746, Val 744, Pro 743, Leu 839, Tyr 32, Leu 13, Ala 16, Val 36, Ala 37
**M2f**		Hydrogen bond interaction: Glu 718, Glu 742 Hydrophobic interaction: Trp 728, Met 746, Phe 712, Val 744, Tyr 32, Pro 743, Val 36, Ala 37, Tyr 837, Leu 839 π–π stacking: Phe 712
**M2g**		Hydrogen bond interaction: Glu 742 Hydrophobic interaction: Pro 717, Phe 838, Leu 839, Phe 840, Met 746, Val 744, Val 36, Pro 743, Leu 13 π–cation interaction: Lys 716
**M2h**		Hydrogen bond interaction: Ser 421, Val 744 Hydrophobic interaction: Tyr 32, Leu 13, Val 36, Ala 37, Ala 16, Pro 717, Met 746, Val 744, Pro 743, Phe 838, Leu 839
**M2i**		Hydrogen bond interaction: Pro 717, Ser 421, Glu 742, Tyr 32, Ash 730, Lys 709 Hydrophobic interaction: Phe 712, Tyr 32, Met 746, Val 36, Val 744, Pro 743, Ala 37, Leu 13, Pro 717
**M3**		Hydrogen bond interaction: Ash 730, Val 744 Hydrophobic interaction: Phe 712, Tyr 32, Pro 717, Val 744, Met 746, Leu 839
**M4**		Hydrogen bond interaction: Tyr 32, Lys 716, Ser 421, Met 746 Hydrophobic interaction: Pro 717, Tyr 417, Tyr 32, Val 36, Val 744, Met 746, Pro 748, Trp 728 π–π stacking: Trp 728 π–cation interaction: Lys 716
**M5**		Hydrogen bond interaction: Val 744, Ash 730, Lys 716, Ser 421 Hydrophobic interaction: Phe 712, Tyr 32, Val 744, Met 746, Pro 717, Phe 838, Leu 839, Phe 840
**M6**		Hydrogen bond interaction: Lys 709, Glu 718, Glu 742 Hydrophobic interaction: Phe 712, Pro 717, Tyr 32, Val 36, Ala 37, Pro 743, Val 744, Met 746, Leu 839, Phe 839, Leu 13 π–cation interaction: Pro 717

**Fig. 7:**
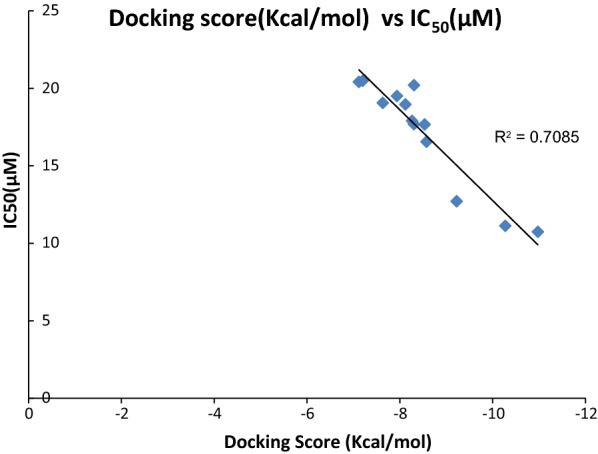
Correlation among docking score and IC_50_ value of synthesized compounds for urease inhibition studies

Hydrogen bond formations were considered as most important for perfect fitting of ligand within the enzyme. Each one of molecule demonstrated good docking score from − 7.117 to − 10.977 as compared to − 3.459 and − 3.049 of standard thiourea and Acetohydroxamic acid as well as excellent binding energy ranges from − 45.27 to − 61.834 kJ/mol as compared to − 21.156 kJ/mol and − 17.454 kJ/mol of standard thiourea and Acetohydroxamic acid. Docking studies concluded that designed ligands have excellent binding capability as compared to parent and standard compounds; a correlation has been set up in docking score and IC_50_ values of synthesized ligands having R^2^ value 0.7085 has been shown in Fig. 7 signfied that molecule having greater the docking score have lesser value of IC_50_ for urease inhibition.

### Admet studies

In drug discovering the ADME profile of drug like molecules is very important and for this purpose Schrodinger’s maestro molecular modeling package Qikprop module was utilized. The Absorption, distribution, metabolism and excretion details of the designed molecules are given in Table 5.

**Table 5:** Simulation studies of derivatives by Quikprop

Synthesised Ligands	QPlogS	QPlog HERG	QPPCaco	QPlogBB	QPlogKp	HBD	HBA	LogP o/w	Rule of Five
M2a	− 5.333	− 6.948	85.491	− 1.979	9.89	5	7	2.629	1
M2b	− 5.373	− 6.405	96.414	− 1.705	9.83	5	7	2.9	1
M2c	− 5.573	− 6.912	9.439	− 3.243	10.254	5	8	1.937	2
M2d	-5.267	− 6.734	8.44	− 3.256	9.974	5	8	1.713	2
M2e	− 5.366	− 6.611	10.739	− 3.046	10.589	5	8	1.933	2
M2f	− 6.14	− 6.647	19.107	− 2.648	10.422	5	8	2.661	3
M2g	− 5.831	− 6.414	13.503	− 2.778	11.635	5	8	2.395	3
M2h	− 4.782	− 6.086	109.727	− 1.611	10.606	5	7	2.657	1
M2i	− 5.646	− 6.361	88.46	− 1.695	9.959	5	7	3.004	2
M3	− 3.201	− 5.146	18.445	− 2.331	11.441	7	8	0.073	1
M4	− 4.438	− 6.8	54.098	− 2.341	10.667	7	8.25	1.869	1
M5	− 5.597	− 8.051	64.909	− 2.614	11.971	5	6.5	2.412	1
M6	− 5.323	− 7.262	20.721	− 3.082	10.963	5	8	2.057	1

Blood brain barrier partition coefficient (QPlogBB), estimated IC_50_ value for HERG K^+^ channels obstruction (log HERG), permeation through skin estimation (QPlogKp), apparent Caco-2 cell permeability estimation in nm/sec (QPPCaco) and apparent MDCK cell permeability estimation in nm/sec (QPPMDCK), partition coefficient in octanol and water Log P, solubility in aqueous media log S, Lipinski’s rule of five. Results revealed that ADME parameters of each ligand within the bounds of satisfactory range without violating Lipinski’s rules.

ADMET calculations showed that synthesised ligands quietly obey rule of five without any considerable violations also many ligands possessed in range values of QPlog S, OPlogHERG, OPPCaco, QPlogBB, QPPMDCK, QPlogKp, QPlogKhsa, HBD, HBA, Log P, % HOA which made them ligands of choice for urease protein [35–39].

## Experimental

### Materials used

Jack bean urease and morin was purchased from Sigma Aldrich and Himedia respectively. Analytical grade reagents and solvents were utilized as a part of study and obtained locally. Progression of reaction was observed via. Thin layer chromatography and recrystallization of products was done in order to purify the compounds which were again checked for purity by thin layer chromatography (TLC) performed on plates covered with silica gel G. Measurement of melting point was done in open capillary tubes on a melting point apparatus and was uncorrected. The spectral data, IR and ^1^H NMR, ^13^CNMR were measured by standard procedures. Brucker 12060280, Germany Software: OPUS 7.2.1394 spectrophotometer in cm^−1^ was used for recording IR spectra of derivatives and elemental analysis was done on Perkin–Elmer 2400 C, H, N analyzer. The ^1^HNMR and ^13^CNMR spectra were recorded in DMSO-d_6_ on a Brucker DRX-300 FTNMR instrument.

### Synthetic procedure

Morin derivatives **(M2a–i, M3–6)** synthesis was accomplished via. a simple and efficient way by two steps which have been outlined in Scheme 1. The synthesis of compouds M2a–M2i was started by formation of substituted aryl thiourea using different substituted anilines then derivatives of morin were prepared reacting synthesized substituted aryl thiourea/thiosemicarbazide/phenylthiosemicarbazide/phenyl hydrazine/benzylcarbazate solution with equimolar concentration of morin in ethanol in presence of acetic acid as catalyst according to previously reported procedure with slight modification [24–26]. Characterisation of all the synthesized compounds was done by IR, ^1^ H NMR, ^13^CNMR and elemental analysis and was found in full accordance with their depicted structures.

### General synthetic procedure for preparation of derivatives of morin

#### Step 1: synthesis of substituted aryl thiourea

Substituted Aniline (0.32 mol) was taken in a 250 ml round bottom flask and, to this, conc. hydrochloric acid (32.19 mL, 0.32 mol) was added dropwise with continues stirring. 100 mL of water was added after appearance of turbidity about after 20 min. followed by addition of ammonium thiocyanate solution (29.42 g, 0.38 mol). This reaction mixture was heated untill the solution becomes turbid, after discontinuing the heating it was poured over ice cold water, and filtration of precipitates was done which were finally dried. Crude product obtained was recrystallized by ethanol.

#### Step 2: synthesis of schiff bases of morin

Equimolar concentration of Morin (0.01 mmol) and substituted arylthiourea/thiosemicarbazide/phenylthiosemicarbazide/benzylcarbazate (0.01 mmol) were solubilized in ethanol (50 ml). Small amount of glacial acetic acid (1–2 ml) was added to the reaction mixture followed by refluxing for 5–6 h. Reaction completion was monitored by thin layer chromatography. Reaction mixture was concentrated; precipitates formed were filtered off and dried. Recrystallization of crude product was done by ethanol and compounds **(M2a–i, M3–6)** were obtained.

##### *N*-*((4E)*-*2*-*(2,4*-*dihydroxyphenyl)*-*3,5,7*-*trihydroxy*-*4H*-*chromen*-*4*-*ylidene)*-*N*-*phenylthiourea* (M2a)

R_f_ = Toluene: Chloroform: methanol (4:4:1) = 0.64; Yield = 71.2%; m.p (246–248 °C); IR cm^−1^ 3649 (O–H str), 3523 (N–Hstr), 1651 (C=Cstr), 1614 (C=N str),1539 (N–C=Sstr), 1313 (C–O str), 1172 (C–N str); ^1^H NMR (400 MHz, DMSO-*d*_6_) δ 12.68 (s, 1H, OH), 10.68 (s, 1H, OH), 9.74 (s, 1H, NH), 9.68 (s, 1H, OH), 9.38 (s, 2H Ar–H), 7.53–7.25 (m, 5H, Ar–H), 7.24 (d, 1H, Ar–H), 7.18–6.90 (m, 2H, Ar–H), 6.14–6.30 (m, 2H, Ar–H); ^13^C NMR (400 MHz, CDCl_3_)δ = 187.81, 166.55, 163.81, 159.55, 157.75, 157.20, 146.11, 139.95, 135.22, 129.13, 127.42–127.13, 123.12, 123.11–122.88, 112.83, 107.64, 104.87, 102.35–102.06, 99.77, 91.34; MS ES + (ToF): m/z 436.07 [M^+^]; CHNS: Calc (C_22_H_16_N_2_O_6_S): C, 60.54; H, 3.70; N, 6.42; S, 7.35; Found: C, 60.57; H, 3.69; N, 6.41; S, 7.33.

##### *N*-*(2*-*chlorophenyl)*-*N*-*((4E)*-*2*-*(2,4*-*dihydroxyphenyl)*-*3,5,7*-*trihydroxy*-*4H*-*chromen*-*4*-*ylidene)thiourea* (M2b)

R_f_ = Toluene: Chloroform: methanol (4:4:1) = 0.66; Yield = 72.8%; m.p (202–204 °C); IR cm^−1^ 3651 (O–H str), 3591 (N–Hstr), 1652 (C=Cstr), 1621 (C=N str), 1541 (N–C=Sstr), 1316 (C–O str), 1174 (C–N str),750 (C–Cl); ^1^H NMR (400 MHz, DMSO-*d*_6_) δ 13.01 (s, 1H, OH), 10.57 (s, 1H, OH), 9.67 (s, 1H, OH), 9.57 (s, 1H, NH), 9.40 (s, 2H, OH), 7.66 (dd, 1H, OH), 7.59–7.33 (m, 3H, Ar–H), 7.05 (m, 1H, Ar–H), 6.62–6.55 (m, 2H, Ar–H), 6.43–6.34 (m, 2H, Ar–H); ^13^C NMR (400 MHz, CDCl_3_)δ = 188.57, 166.92, 163.11, 159.53, 157.93, 157.18, 142.31, 136.37, 135.13, 130.75–129.18, 128.20, 127.89–127.74, 127.28, 126.07, 124.72–124.43, 113.43, 107.63, 104.85, 101.20–101.07, 97.68, 91.51; MS ES + (ToF): m/z 470.02 [M^+^]; CHNS: Calc (C_22_H_15_ClN_2_O_6_S): C, 56.11; H, 3.21; N, 5.95; S, 6.81; Found: C, 56.09; H, 3.22; N, 5.94; S, 6.80.

##### *N*-*((4E)*-*2*-*(2,4*-*dihydroxyphenyl)*-*3,5,7*-*trihydroxy*-*4H*-*chromen*-*4*-*ylidene)*-*N*-*(2*-*nitrophenyl)thiourea* (M2c)

R_f_ = Toluene: Chloroform: methanol (4:4:1) = 0.62; Yield = 65.7%; m.p (238–240 °C); IR cm^−1^ 3670 (O–H str), 3565 (N–Hstr), 1652 (C=Cstr), 1625 (C=N str), 1571 (N–C=Sstr), 1508 (N–O str), 1344 (C–O str), 1170 (C–N str); ^1^H NMR (400 MHz, DMSO-*d*_6_) δ 12.83 (s, 1H, OH), 10.44 (s, 2H, OH), 9.88 (s, 1H, OH), 9.59 (s, IH, NH), 9.42 (s, 2H, OH), 8.50 (dd, 1H, Ar–H), 8.28 (dd, 1H, Ar–H), 7.71 (td, 1H, Ar–H), 7.37–7.28 (m, 2H, Ar–H), 6.63–6.55 (m, 2H, Ar–H), 6.43–6.34 (m, 2H, Ar–H); ^13^C NMR (400 MHz, CDCl_3_)δ = 187.91, 166.57, 163.81, 159.94, 157.93, 157.19, 145.11, 137.36, 136.04, 135.18, 132.22, 127.90–127.67, 126.54–126.25, 125.44–125.14, 118.88, 112.43, 107.62, 104.85, 102.36–102.10, 99.68, 91.73; MS ES + (ToF): m/z 481.06 [M^+^]; CHNS: Calc (C_22_H_15_N_3_O_8_S): C, 54.88; H, 3.14; N, 8.73; S, 6.66; Found: C, 54.85; H, 3.16; N, 8.72; S, 6.66.

##### *N*-*((4E)*-*2*-*(2,4*-*dihydroxyphenyl)*-*3,5,7*-*trihydroxy*-*4H*-*chromen*-*4*-*ylidene)*-*N*-*(3*-*nitrophenyl)thiourea* (M2d)

R_f_ = Toluene: Chloroform: methanol (4:4:1) = 0.63; Yield = 71.9%; m.p (240–242 °C); IR cm^−1^ 3690 (O–H str), 3549 (N–Hstr), 1698 (C=Cstr), 1614 (C=N str),1541 (N–C=Sstr), 1507 (N–O) 1302 (C–O str), 1172 (C–N str); ^1^H NMR (400 MHz, DMSO-*d*_6_) δ 13.05 (s, 1H, OH), 10.48 (s, 1H, OH), 9.87 (s, 1H, OH), 9.65 (s, 1H, NH), 9.41 (s, 2H, OH), 8.54–8.48 (m, 1H, Ar–H), 7.96–7.86 (m, 2H, Ar–H), 7.55 (m, 1H, Ar–H), 7.34 (d, 1H, Ar–H), 6.60–6.52 (m, 2H, Ar–H), 6.54–6.36 (m, 2H, Ar–H); ^13^C NMR ^13^C NMR (400 MHz, CDCl_3_)δ = 187.75, 166.57, 163.81, 159.94, 157.93, 157.19, 148.76, 145.11, 140.08, 135.18, 131.13, 128.33, 127.88–127.79, 117.85, 116.90, 112.43, 107.62, 104.85, 102.38–102.10, 99.67, 91.73; MS ES + (ToF): m/z 481.9 [M^+^]; CHNS: Calc (C_22_H_15_N_3_O_8_S): C, 54.88; H, 3.14; N, 8.73; S, 6.66; Found: C, 54.86; H, 3.13; N, 8.75; S, 6.65.

##### *N*-*((4E)*-*2*-*(2,4*-*dihydroxyphenyl)*-*3,5,7*-*trihydroxy*-*4H*-*chromen*-*4*-*ylidene)*-*N*-*(4*-*nitrophenyl)thiourea* (M2e)

R_f_ = Toluene: Chloroform: methanol (4:4:1) = 0.65; Yield = 69.4%; m.p (240–242 °C); IR cm^−1^ 3650 (O–H str), 3523 (N–Hstr), 1656 (C=Cstr), 1611 (C=N str), 1539 (N–C=Sstr), 1508 (N–O), 1307 (C–O str), 1173 (C–N str); ^1^H NMR (400 MHz, DMSO-*d*_6_) δ 13.08 (s, 1H, OH), 11.61 (s, 1H, OH), 11.14 (s, 1H, OH), 10.40 (s, 1H, OH), 9.88 (s, 1H, NH), 8.34–8.26 (m, 2H, Ar–H), 7.78–7.70 (m, 2H, Ar–H), 7.34 (d, 1H, Ar–H), 6.64–6.57 (m, 2H, Ar–H), 6.41–6.32 (m, 2H, Ar–H); ^13^C NMR (400 MHz, CDCl_3_)δ = 187.83, 166.57, 163.81, 159.94, 157.93, 157.18, 145.11, 143.20, 135.18, 127.95–127.69, 125.73–125.44, 121.40, 112.43, 107.62, 104.85, 102.36–102.09, 99.66, 91.74; MS ES + (ToF): m/z 481.09[M^+^]; CHNS: Calc (C_22_H_15_N_3_O_8_S): C, 54.88; H, 3.14; N, 8.73; S, 6.66; Found: C, 54.86; H, 3.15; N, 8.72; S, 6.67.

##### *N*-*(4*-*chloro*-*2*-*nitrophenyl)*-*N*-*((4E)*-*2*-*(2,4*-*dihydroxyphenyl)*-*3,5,7*-*trihydroxy*-*4H*-*chromen*-*4*-*ylidene)thiourea* (M2f)

R_f_ = Toluene: Chloroform: methanol (4:4:1) = 0.69; Yield = 68.5%; m.p (228–230 °C); IR cm^−1^ 3669 (O–H str), 3587 (N–Hstr), 1655 (C=Cstr), 1627 (C=N str),1540 (N–C=Sstr), 1506 (N–O str), 1295 (C–O str), 1174 (C–N str), 791 (C–Cl); ^1^H NMR (400 MHz, DMSO-*d*_6_) δ 13.15 (s, 1H, OH), 12.99 (s, 1H, NH), 11.45 (s, 1H, OH), 11.14 (s, 1H, OH), 10.44 (s, 1H, OH), 9.86 (s, 1H, OH), 8.08 (s, 1H, Ar–H), 7.79 (d, 1H, Ar–H), 7.55 (d, 1H, Ar–H), 7.29 (d, 1H, Ar–H), 6.62–6.55 (m, 2H, Ar–H), 6.41–6.32 (m, 2H, Ar–H); ^13^C NMR (400 MHz, CDCl_3_)δ = 187.91, 166.57, 163.81, 159.94, 157.93, 157.18, 145.11, 136.04, 135.18, 133.46, 127.95–127.66, 127.51–127.20, 125.21, 112.43, 109.18–30.08; MS ES + (ToF): m/z 515.02 [M^+^]; CHNS: Calc (C_22_H_14_ClN_3_O_8_S): C, 51.22; H, 2.74; N, 8.15; S, 6.22; Found: C, 51.24; H, 2.75; N, 8.12; S, 6.20.

##### *N*-*(2*-*chloro*-*4*-*nitrophenyl)*-*N*-*((4E)*-*2*-*(2,4*-*dihydroxyphenyl)*-*3,5,7*-*trihydroxy*-*4H*-*chromen*-*4*-*ylidene)thiourea* (M2 g)

R_f_ = Toluene: Chloroform: methanol (4:4:1) = 0.61; Yield = 70.4%; m.p (226–228 °C); IR cm^−1^ 3649 (O–H str), 3547 (N–Hstr), 1650 (C=Cstr), 1616 (C=N str),1524 (N–C=Sstr), 1502 (N–O str),1304 (C–O str), 1173 (C–N str), 814 (C–Cl str); ^1^H NMR (400 MHz, DMSO-*d*_6_) δ 13.19 (s, 1H, OH), 12.41 (s, 2H, NH), 11.45 (s, 1H, OH), 10.44 (s, 1H, OH), 9.88 (s, 1H, OH), 8.24 (d, 1H, OH), 8.07 (dd, 1H, Ar–H), 7.81 (d, 1H, Ar–H), 7.29 (d, 1H, Ar–H), 6.67–6.55 (m, 2H, Ar–H), 6.41–6.32 (m, 2H, Ar–H); ^13^C NMR (400 MHz, CDCl_3_)δ = 188.48, 166.57, 163.81, 159.94, 157.93, 157.18, 145.11, 140.54, 139.78, 135.18, 127.92–127.69, 126.10–125.82, 124.63, 123.27–123.01, 122.80, 112.43, 107.62, 104.85, 102.38–102.07, 99.67, 91.74; MS ES + (ToF): m/z 515.22 [M^+^]; CHNS: Calc (C_22_H_14_ClN_3_O_8_S): C, 51.22; H, 2.74; N, 8.15; S, 6.22; Found: C, 51.24; H, 2.75; N, 8.12; S, 6.20.

##### *N*-*((4E)*-*2*-*(2,4*-*dihydroxyphenyl)*-*3,5,7*-*trihydroxy*-*4H*-*chromen*-*4*-*ylidene)*-*N*-*(2*-*fluorophenyl)thiourea* (M2 h)

R_f_ = Toluene: Chloroform: methanol (4:4:1) = 0.71; Yield = 74.2%; m.p (206–208 °C); IR cm^−1^ 3648 (O–H str), 3529 (N–Hstr), 1651 (C=Cstr), 1614 (C=N str), 1539 (N–C=Sstr), 1313 (C–O str), 1204 (C–F str), 1172 (C–N str); ^1^H NMR (400 MHz, DMSO-*d*_6_) δ 13.16 (s, 1H, OH), 11.49 (s,1H, OH), 11.16 (s, 1H, OH), 10.61 (s, 1H, NH), 10.39 (s, 1H, OH), 9.80 (s, 1H, OH), 7.95 (m, 1H, Ar–H), 7.30 (d, 1H, Ar–H), 7.22–6.98 (m, 4H, Ar–H), 6.61–6.56 (m, 2H, Ar–H), 6.41–6.32 (m, 2H, Ar–H); ^13^C NMR(400 MHz, CDCl_3_)δ = 188.46, 166.57, 163.81, 159.94, 157.93, 157.18, 145.11, 135.18, 129.03–62.61; MS ES + (ToF): m/z 545.06 [M^+^]; CHNS: Calc (C_22_H_15_FN_2_O_6_S): C, 58.15; H, 3.33; N, 6.16; S, 7.06; Found: C, 58.12; H, 3.34; N, 6.14; S, 7.08.

##### *N*-*(4*-*bromophenyl)*-*N*-*((4E)*-*2*-*(2,4*-*dihydroxyphenyl)*-*3,5,7*-*trihydroxy*-*4H*-*chromen*-*4*-*ylidene)thiourea* (M2i)

R_f_ = Toluene: Chloroform: methanol (4:4:1) = 0.74; Yield = 67.3%; m.p (222–224 °C); IR cm^−1^ 3628 (O–H str), 3566 (N–Hstr), 1684 (C=Cstr), 1616 (C=N str),1508 (N–C=Sstr), 1369 (C–O str), 1189 (C–N str), 613 (C–Br str); ^1^H NMR (400 MHz, DMSO-*d*_6_) δ 13.11 (s, 1H, OH), 11.45 (s, 1H, OH), 11.34 (s, 1H, NH), 11.14 (s, 1H, OH), 10.47 (s, 1H, OH), 9.88 (s, 1H), 7.56–7.44 (m, 4H, Ar–H), 7.34 (d, 1H, Ar–H), 6.62–6.55 (m, 2H, Ar–H), 6.43–6.34 (m, 2H, Ar–H); ^13^C NMR (400 MHz, CDCl_3_)δ = 230.03–126.59 (m), 126.59–25.04 (m); MS ES + (ToF): m/z 515.98 [M^+^]; CHNS: Calc (C_22_H_15_BrN_2_O_6_S): C, 51.27; H, 2.93; N, 5.44; S, 6.22; Found: C, 51.25; H, 2.90; N, 5.47;; S, 6.20.

##### *(1Z)*-*1*-*(3,5,7*-*trihydroxy*-*2*-*(2,4*-*dihydroxyphenyl)*-*4H*-*chromen*-*4*-*ylidene)thiosemicarbazide* (M3)

R_f_ = Toluene: Chloroform: methanol (4:4:1) = 0.68; Yield = 68.8%; m.p (204–206 °C); IR cm^−1^ 3631 (O–H str), 3521 (N–Hstr), 1650 (C=Cstr), 1623 (C=N str), 1539 (N–C=Sstr), 1319 (C–O str), 1225 (C–N str), 1033 (N–N str); ^1^H NMR (400 MHz, DMSO-*d*_6_) δ 12.21 (s, 1H, OH), 11.70 (s, 1H, NH), 10.12 (s, 1H, OH), 9.95 (s, 1H, OH), 9.88 (s, 1H),9.54 (s, OH, 1H), 8.64 (s, 2H, NH_2_), 7.59 (t, 1H, Ar–H), 6.59–6.51 (m, 2H, Ar–H), 6.43–6.27 (m, 2H, Ar–H); ^13^C NMR (400 MHz, CDCl_3_)δ = 180.16, 163.73, 159.94, 157.11, 155.70, 154.25, 143.84, 131.07, 130.47, 127.92–127.71, 111.89, 107.62, 102.38–102.09, 101.83, 99.06, 91.12; MS ES + (ToF): m/z 375.05 [M^+^]; CHNS: Calc (C_16_H_13_N_3_O_6_S): C, 51.20; H, 3.49; N, 11.19; S, 8.54; Found: C, 51.20; H, 3.49; N, 11.19; S, 8.54.

##### *(4Z)*-*4*-*(3,5,7*-*trihydroxy*-*2*-*(2,4*-*dihydroxyphenyl)*-*4H*-*chromen*-*4*-*ylidene)*-*1*-*phenylthiosemicarbazide* (M4)

R_f_ = Toluene: Chloroform: methanol (4:4:1) = 0.72; Yield = 73.1%; m.p (198–200 °C); IR cm^−1^ 3616 (O–H str), 3562 (N–Hstr), 1651 (C=Cstr), 1601 (C=N str), 1521 (N–C=Sstr), 1338 (C–O str), 1186 (C–N str), 1062 (N–N str); ^1^H NMR (400 MHz, DMSO-*d*_6_) δ 13.21 (s, 1H, OH), 11.45 (s, 1H, OH), 10.44 (s, 1H), 9.85 (s, 1H), 9.68 (s, 1H, OH), 9.46 (s, 1H, OH), 7.34 (d, 1H, Ar–H), 7.23–7.13 (m, 2H, Ar–H), 7.07–6.99 (m, 2H, Ar–H), 6.86 (t, 1H, Ar–H), 6.62–6.55 (m, 2H, Ar–H), 6.43–6.34 (m, 2H), 5.70 (d, 1H, Ar–H); ^13^C NMR (400 MHz, CDCl_3_)δ = 182.86, 169.47, 163.81, 159.94, 157.93, 157.18, 147.22, 145.11, 135.18, 128.66, 127.95–127.69, 120.66, 114.89, 112.43, 107.62, 104.85, 102.38–102.07, 99.67, 91.74; MS ES + (ToF): m/z 451.08 [M^+^]; CHNS: Calc (C_22_H_17_N_3_O_6_S): C, 58.53; H, 3.80; N, 9.31; S, 7.10; Found: C, 58.50; H, 3.82; N, 9.30; S, 7.09.

##### *(4Z)*-*2*-*(2,4*-*dihydroxyphenyl)*-*4*-*(2*-*phenylhydrazinylidene)*-*4H*-*chromene*-*3,5,7*-*triol* (M5)

R_f_ = Toluene: Chloroform: methanol (4:4:1) = 0.75; Yield = 62.8%; m.p (210–212 °C); IR cm^−1^ 3639 (O–H str), 3568 (N–Hstr), 1652 (C=Cstr), 1570 (C=N str), 1363 (C–O str), 1163 (C–N str), 1042 (N–N)str); ^1^H NMR (400 MHz, DMSO-*d*_6_) δ 12.24 (s, 1H, OH), 11.42 (s, 1H, OH), 11.10 (s, 1H, OH), 10.44 (s, 1H, NH), 9.86 (s, 1H, OH), 9.72 (s, 1H, OH), 7.50– 7.42 (m, 2H), 7.33–7.24 (m, 2H, Ar–H), 7.09–7.00 (m, 1H, Ar–H), 6.58–6.51 (m, 2H, Ar–H), 6.40–6.30 (m, 2H, Ar–H); ^13^C NMR (400 MHz, CDCl_3_)δ = 163.73, 159.94, 157.11, 155.69, 154.25, 143.84, 143.62, 131.08, 130.65, 128.97, 127.93–127.69, 119.95, 116.25–115.92, 111.89, 107.62, 102.38–102.09, 101.86, 99.06, 91.12; MS ES + (ToF): m/z 392.10 [M^+^]; CHNS: Calc (C_21_H_16_N_2_O_6_): C, 64.28; H, 4.11; N, 7.14; Found: C, 64.26; H, 4.12; N, 7.13.

##### *(4Z)*-*2*-*(2,4*-*dihydroxyphenyl)*-*4*-*(2*-*phenylhydrazinylidene)*-*4H*-*chromene*-*3,5,7*-*triol* (M6)

R_f_ = Toluene: Chloroform: methanol (4:4:1) = 0.77; Yield = 58.6%; m.p (250–252 °C); IR cm^−1^ 3670 (O–H str), 3520 (N–H str), 1651 (C=C str), 1618 (C=N str), 1338 (C–O str), 1170 (C–N str), 1078 (N–N str); ^1^H NMR (400 MHz, DMSO-*d*_6_) δ 14.19 (s, 1H, NH), 11.45 (s, 2H, OH), 11.14 (s, 1H, OH), 10.49 (s, 1H, OH), 9.88 (s, 1H, OH), 9.70 (s, 1H, OH), 7.39–7.24 (m, 6H, Ar–H), 6.57 (dd, 2H, Ar–H), 6.43–6.34 (m, 2H, Ar–H), 5.21 (d, 2H, Ar–H); ^13^C NMR (400 MHz, CDCl_3_)δ = 163.73, 159.94, 157.11, 155.70, 154.25, 154.02, 143.84, 137.12, 131.07, 130.71, 128.64–113.49, 111.89, 107.62, 102.40–102.07, 101.68, 99.06, 91.12, 66.08; MS ES + (ToF): m/z 450.11 [M^+^]; CHNS: Calc (C_23_H_18_N_2_O_8_): C, 61.33; H, 4.03; N, 6.22; Found: C, 61.35; H, 4.01; N, 6.24.

### *In-silico study protocol*

In-silico studies were done to study the interaction pattern of newly designed ligands in the catalytic cavity of jack bean urease enzyme. X-ray crystal structure of Jack Bean Urease enzyme having resolution 2.05 Å was downloaded from Protein Data Bank (http://www.rcsb.org) (PDB code: 3LA4) from the Research Collaboratory for Structural bioinformatics (RCSB). Structure of protein was prepared first of all before docking studies by using pre-process method of Prepwiz module of Schrodinger’s Maestro molecular modeling. Bond orders were assigned, partial charges and hydrogen were added, side chains and loops having missing atoms were fabricated, all waters (with exception of those which were coordinated to metals) were deleted. Structure of proposed Ligands was drawn using Chemdraw Ultra 8.0 (ChemOffice Package) software which was saved as mol. file. Preparation of ligand was done prior to docking by Ligprep module of Schrodinger’s Maestro molecular modeling. Optimization of structure was done by the Gaussian 03 package. Ideal conformation of ligands was acquired by the adding or removing hydrogen bonds, computing partial charges according to the OPLS-2005 force field with 32 stereo isomers, tautomers, and other options like ionization at the physiological pH 7.2 were set as default options. Ligands each conformation was docked within radii of 20 Å into the receptor lattice at the coupling destinations of receptor structures as per their minimum potential energy. Glide/XP by default joined with Induced Fit docking conventions was connected for the forecast of energy of binding and interaction within protein and ligand at the catalytic sites of enzyme. Induced Fit Docking (IFD) methods give the springiness to ligand as well as the residues present at active site all over the docking study where it was rotated in all three angles [37–40]. Finally, the result in Glide score (*GScore*) scoring function was obtained as the output for each ligand given in Table 3.

### Urease inhibitiory assay

Urease enzyme inhibition investigation studies for all synthesized compounds were done by the method developed by Weatherburn [27] named Indophenol against Jack Bean Urease. Briefly, incubation of solution of Jack bean protein 25 *μ*L was done at temperature of 30 °C for a period of 15 min with 55 *μ*L of buffers solution having 100 mM urea and 5 *μ*L of test solution in 96-well plates. 45 *μ*L phenol solution composing phenol 1% w/v and solution of sodium nitroprusside 0.005% w/v and alkali reagent 70 *μ*L composed of sodium hydroxide 0.5% w/v and active sodium hypochloride solution 0.1% were mixed to every well. The measurement of increase in absorption at 625 nm was done after 50 min by help of a microplate reader (Molecular Device, USA). Readings were recorded in triplicate set in a final volume of 200 *μ*L using thiourea as standard. Each assay was performed at pH 8.2 (0.01 M K_2_HPO_4_.3H_2_O, 0.01 M LiCl_2_ and 1 mM EDTA). Calculation of Inhibition in percentage of synthesized derivatives was done by the formula given as:

$${\text{I\% = }}\frac{{{\text{A}}\,{\text{Control}} - {\text{A}}\,{\text{Sample}}}}{{{\text{A}}\,{\text{Control}}}} \times 100$$

where, A_control_ is control absorbance; A_sample_ is test absorbance

### Dpph assay for antioxidant activity

DPPH method was adopted for measuring the antioxidant nature of the synthesized derivatives. According to this method synthesized compounds were allowed to react for 0.5 h. at 37 °C with stable free radical, 1, 1-diphenyl-2-picrylhydrazyl radical (DPPH). The DPPH was taken in a concentration of 300 µM. Decrease in value of absorption at 515 nm was measured after the period of incubation using a microplate reader using ascorbic acid as a standard. Molecule which can donate a proton to DPPH and cause its reduction act as an antioxidant. Following reduction deep violet coloured DPPH solution changes to yellow depending upon the nature of antioxidant compound, which brings a measurable decrease in value of absorption at 517 nm. The proton or electron donating capacity of the derivative was estimated from the fading of deep purple colored methanolic solution of 1, 1-diphenylpicrylhydrazyl (DPPH). Incubation of mixture was done at room temperature for 30 min and measurements were done at 517 against blank [28, 29]. Calculation of percentage inhibition was done by following formula:

$${\text{I\% = }}\frac{{{\text{A}}\,{\text{Control}} - {\text{A}}\,{\text{Sample}}}}{{{\text{A}}\,{\text{Control}}}} \times 100$$

where, A_control_ is control absorbance; A_sample_ is test absorbance

### Anti-*H. pylori* activity

Novel synthesized derivatives were investigated against *H. pylori* bacterium (ATCC 43504 DSM 4867, AHA as standard) for their antibacterial activity by using Well diffusion technique. The stock solution of compounds in DMSO (1000 μg/mL) were prepared. Cell suspension was prepared from culture grown on BHI broth. The cell suspensions of all the cultures were adjusted to 1–2 × 10^5^cells/ml. *Helicobacter pylori* (100 µl) was inoculated by spread plate technique on agar plates (90 mm). Agar wells (5 mm) were made on *H. pylori* inoculated media and impregnated with 2500 µg of each sample and standard which were incubated @ 35 °C for 24–48 h with 5% CO_2_ and observed for zone of inhibition around the well. MIC was determined against *Helicobacter pylori* by micro broth dilution technique as per NCCLS method [41–45].

### Kinetic parameters of urease activity

Kinetic parameters i.e. Km, Vmax and Michaelis constant were calculated by verifying the concentration effect of substrate on the rate of reaction of urease enzyme in presence as well as in absence of synthesized compounds. Mechanism behavior of derivatives was studied by dissolving 1% of them in dimethyl sulfoxide and inhibition mode studied by Lineweaver–Burk plots was found to be non-competitive as Km was contant but Vmax changed. Urease enzyme kinetic studies of interaction with synthesized was done GraphPad Prism 7 software.

### Statistical analysis

Output of statistical analysis has been depicted in mean ± SEM whereas statistical examination of data collected experimentally was done by one-way analysis of variance (ANOVA). Considerable difference revealed by ANOVA p < 0.05 was regarded significant. Evaluation of statistical data was done by Graph Pad Prism 5.0 Version for Windows (San Diego, CA, USA).

## Conclusion

In conclusion, thirteen Morin derivatives have been synthesized successfully via. a simple two step reaction and evaluated for their DPPH- free radical scavenging activity and urease inhibitory activity against jack bean urease. Among the series M2b, M2i and M2a (IC_50_ 110.74 ± 0.018, 11.12 ± 0.033 and 12.71 ± 0.027 µM even two folds more active than standard drug thiourea and also displayed good antioxidant behavior with IC_50_ value 7.37 ± 0.024, 7.73 ± 0.015 and 7.795 ± 0.003 µM. Moreover these are found to be most potent by having excellent dock score 10.977, − 10.273, − 9.225 and binding energy − 59.062, − 45.27, − 61.834 kJ/mol as compared to standard drugs − 3.459, − 3.049 and − 21.156 kJ/mol and − 17.454 kJ/mol of standard thiourea and Acetohydroxamic acid. These top molecules were again further examined for anti-*H. Pylori* activity and M2i was found to be more potent as compared to standard drug AHA with MIC = 500 *μ*g/ml and zone of inhibition 15.00 ± 0.00 mm as compared to standard having MIC = 1000 *μ*g/ml and zone of inhibition 9.00 ± 0.50 mm. Derivative M2i can be the potential lead compound in future for treatment of pathologies caused by urease as well as against *H. Pylori* infection.

## Additional file

**Additional file 1.** Supplementary file for spectral data.

## Abbreviations

ADMET: absorption distribution metabolism excretion
MIC: minimum inhibitory concentration
TLC: thin layer chromatography
UV: ultra violet
DPPH: 2, 2-diphenyl-1-picrylhydrazyl
SEM: standard error mean
ANOVA: analysis of variance
DMSO: dimethyl sulphoxide
AHA: acetohydroxamic acid
HOA: human oral absorption
RCSB: Research Collaboratory for Structural bioinformatics
NCCLS: National Committee for Clinical Laboratory Standards
