# Supplementary material for: Molecular docking, synthesis, kinetics study, structure–activity relationship and ADMET analysis of morin analogous as Helicobacter pylori urease inhibitors
Source: BMC Chem. 2019 Apr 1;13(1):45. doi: 10.1186/s13065-019-0562-2 (PMC6661831; doi:10.1186/s13065-019-0562-2)
Supplement: Supplementary file 1 — Additional file 1. Supplementary file for spectral data. [file 13065_2019_562_MOESM1_ESM.docx]

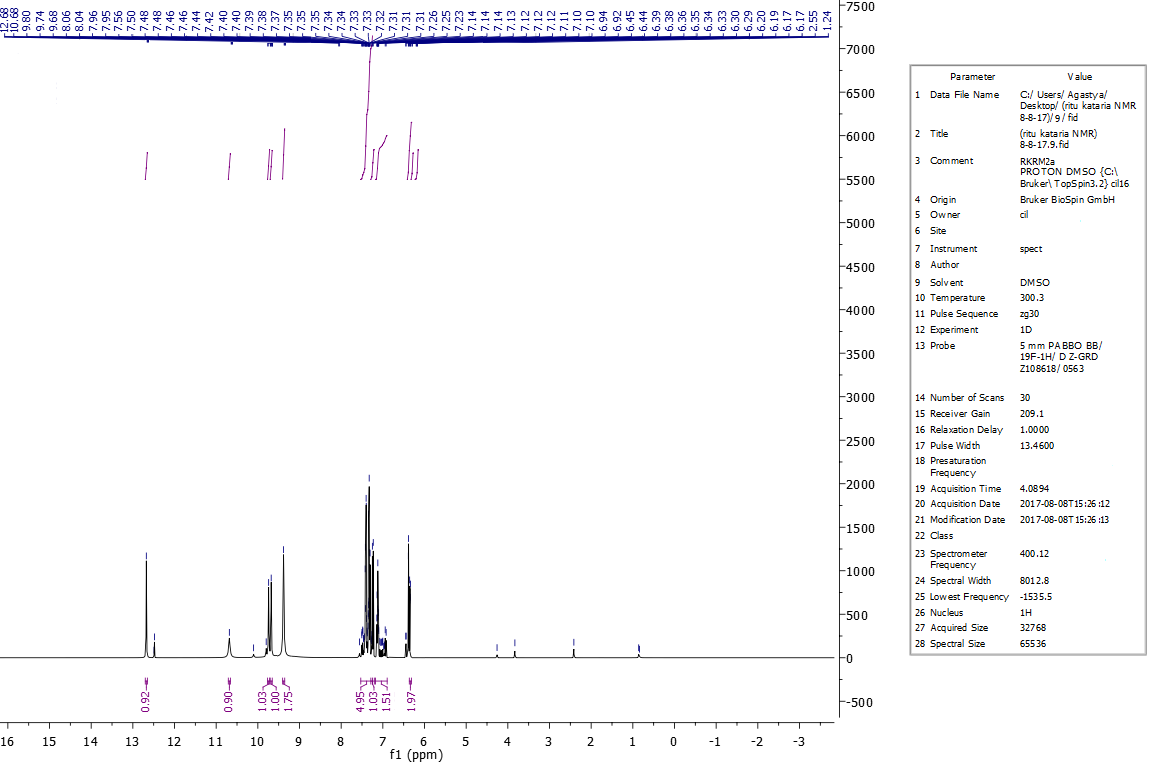


^1^HNMR of Compound M2a


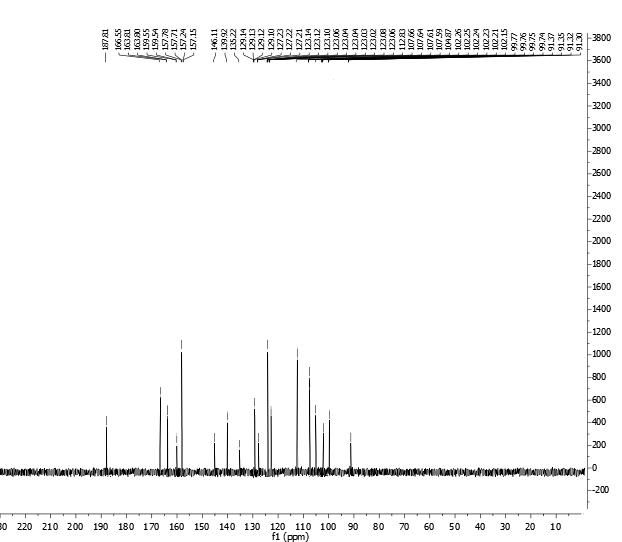


^13^CNMR of Compound M2a


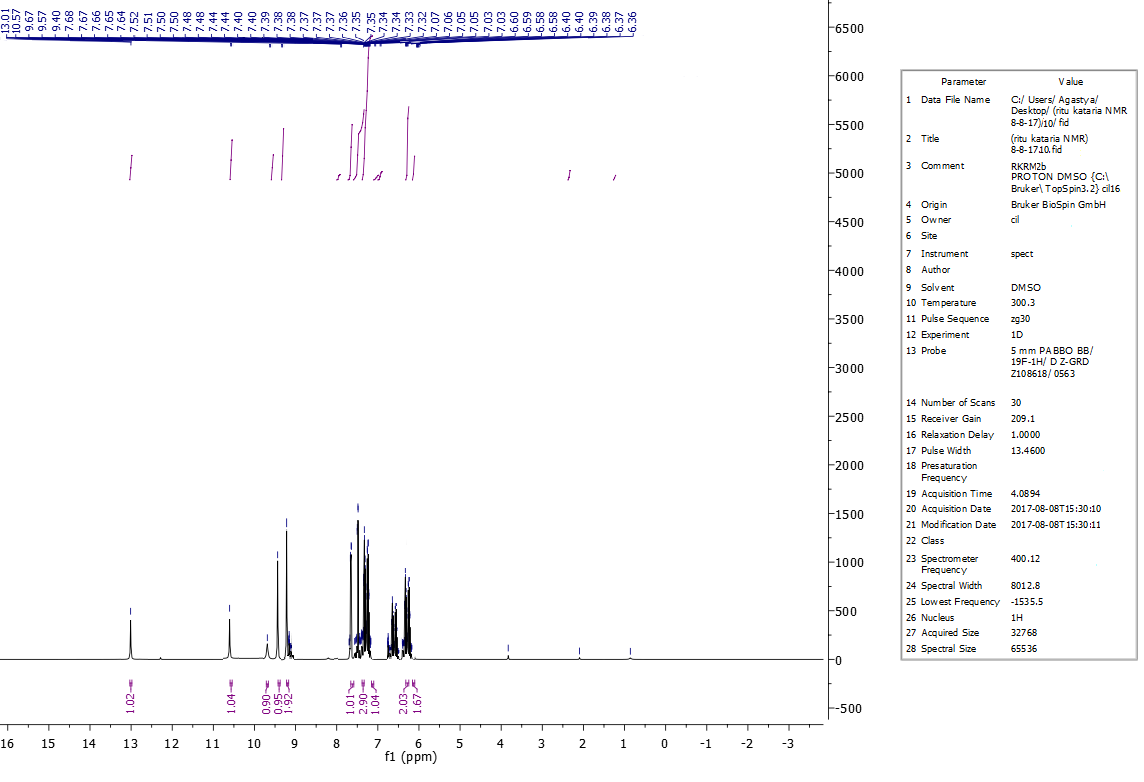


^1^HNMR of Compound M2b


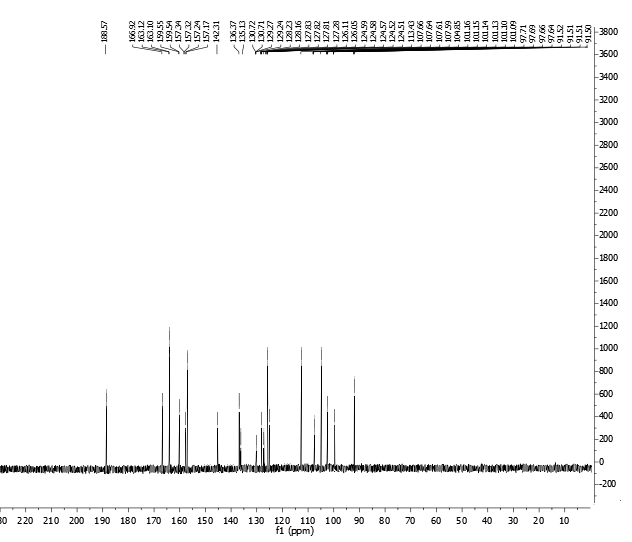


^13^CNMR of Compound M2b


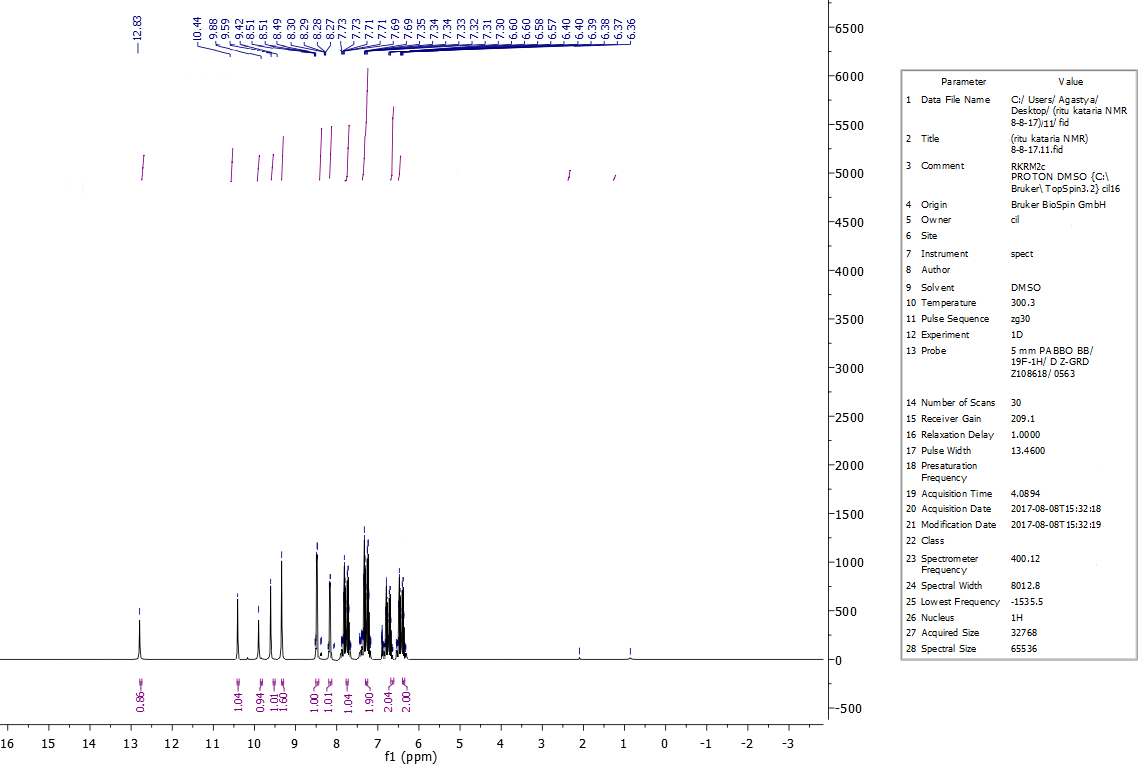


^1^HNMR of Compound M2c


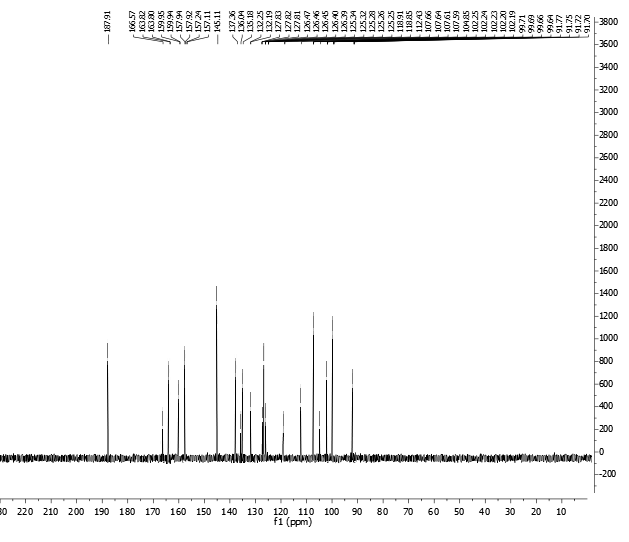


^13^CNMR of Compound M2c


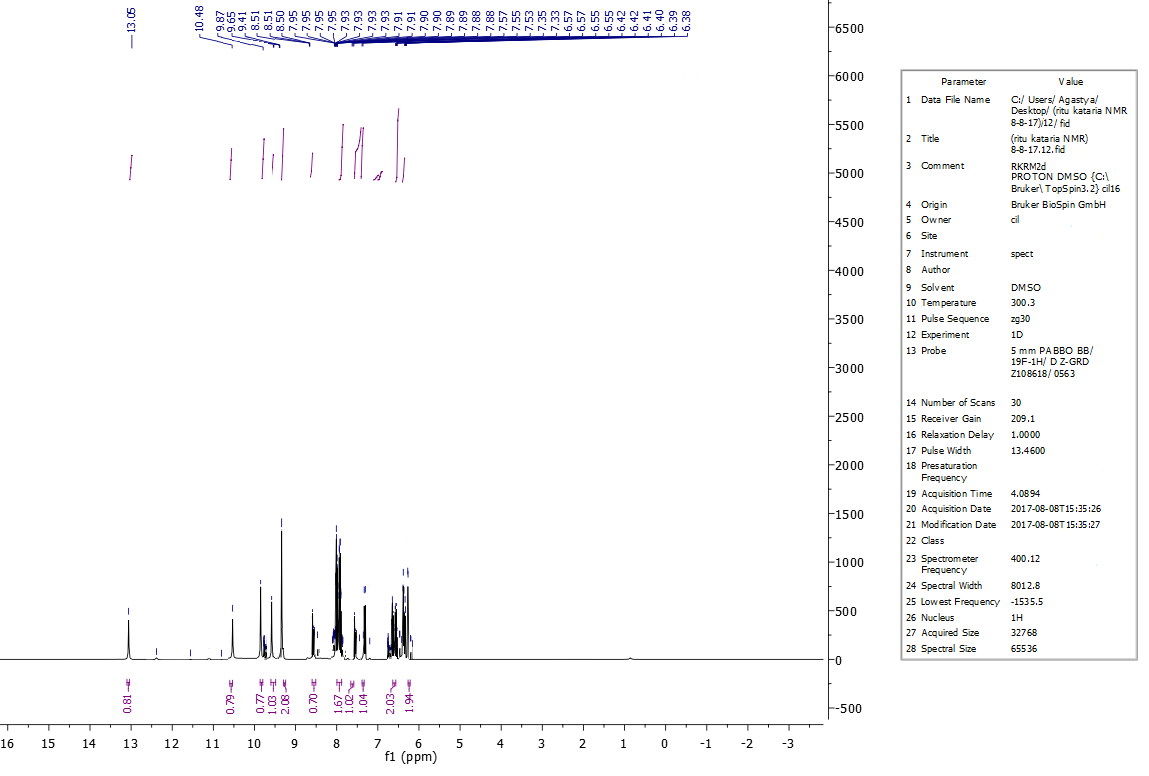


^1^HNMR of Compound M2d


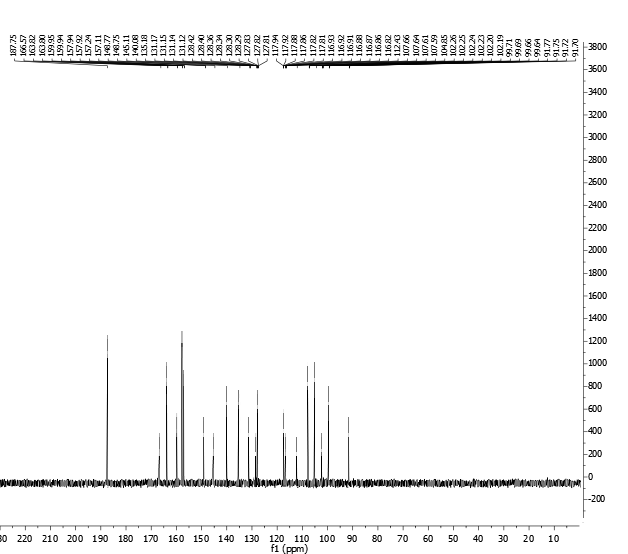


^13^CNMR of Compound M2d


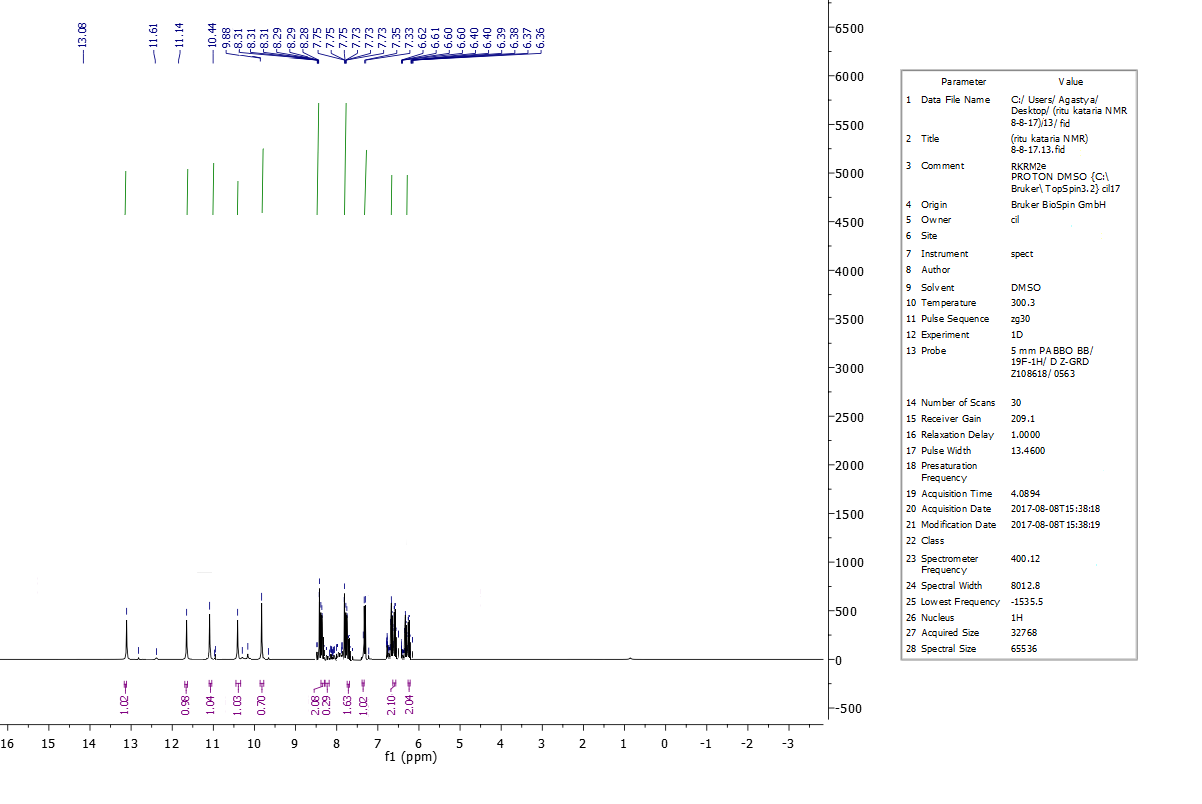


^1^HNMR of Compound M2e


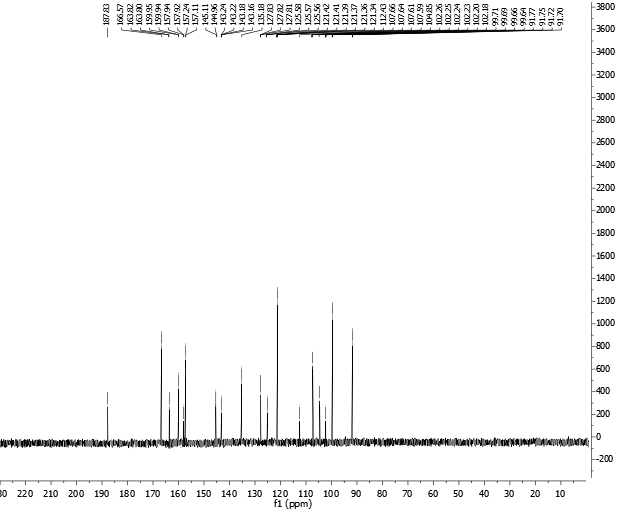


^13^CNMR of Compound M2e


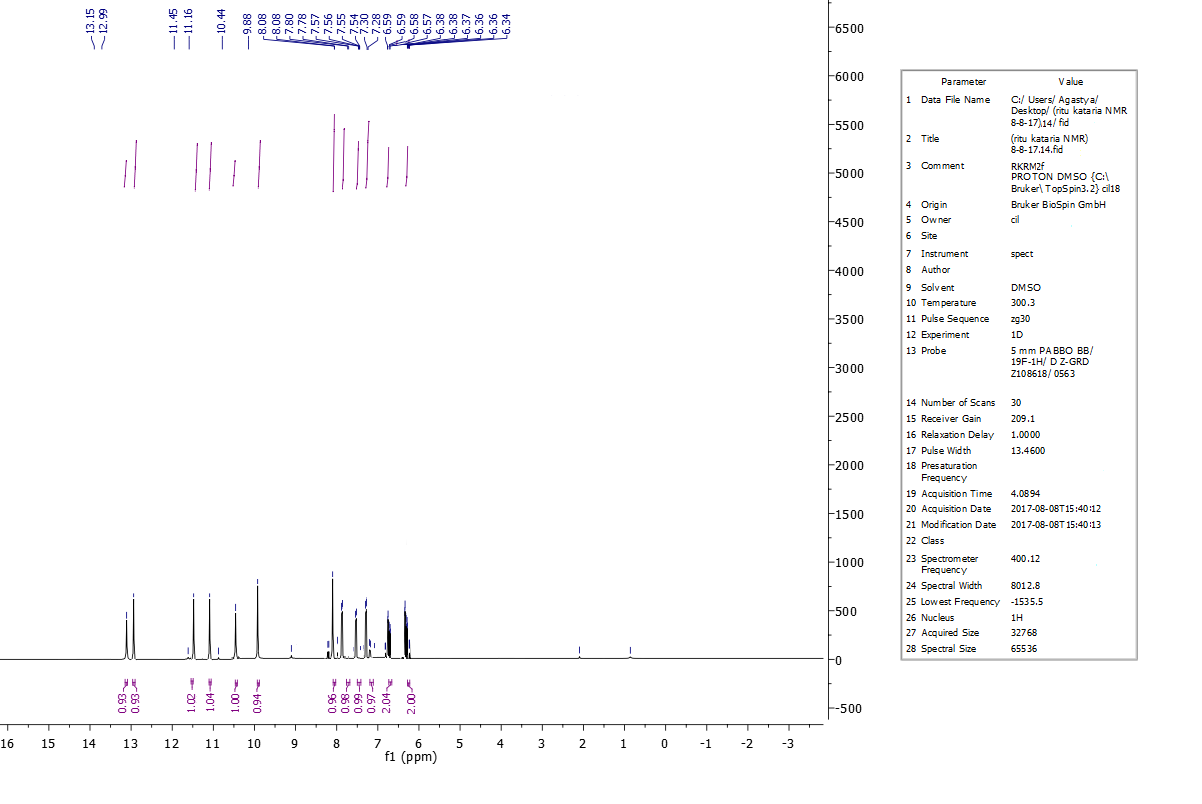


^1^HNMR of Compound M2f


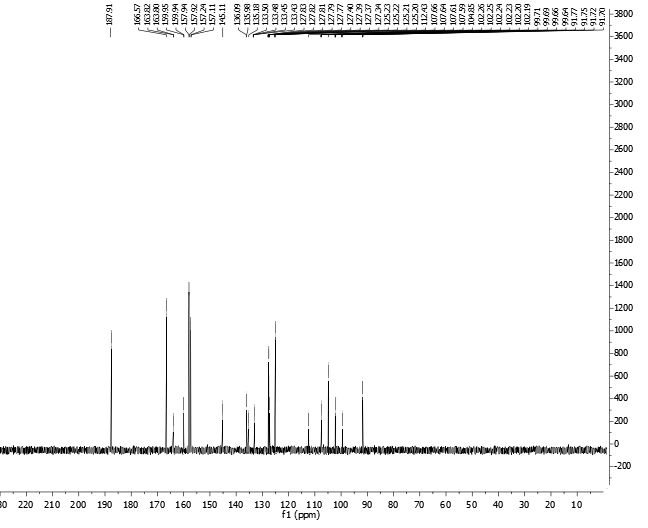


^13^CNMR of Compound M2f


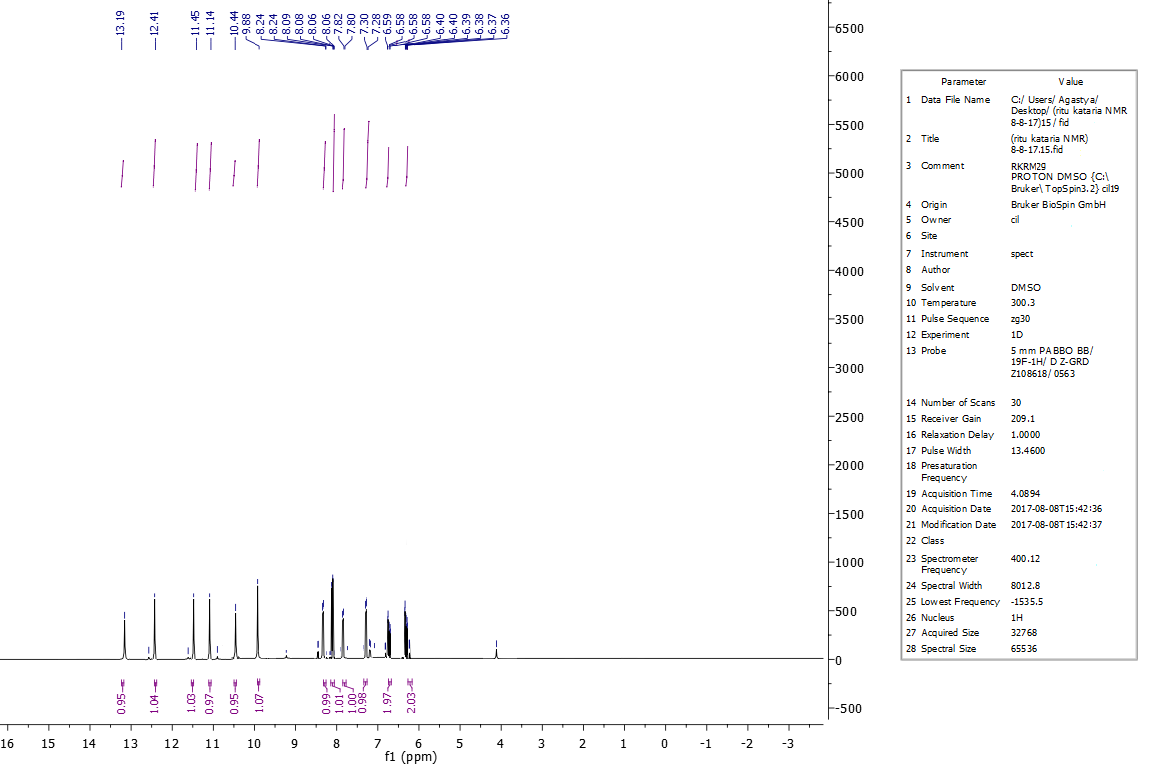


^1^HNMR of Compound M2g


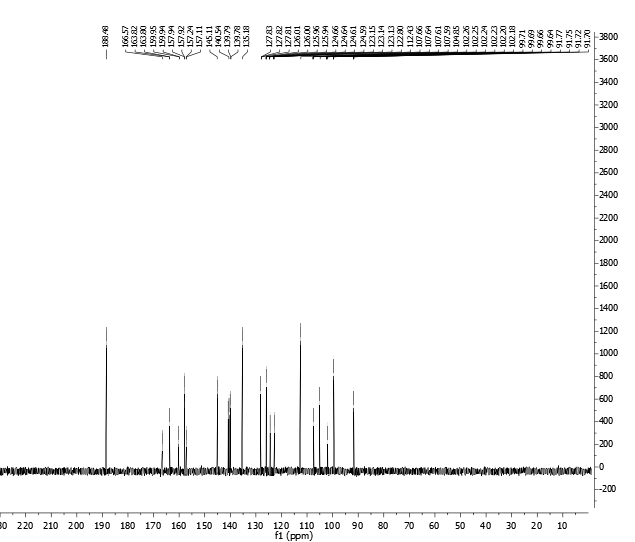


^13^CNMR of Compound M2g


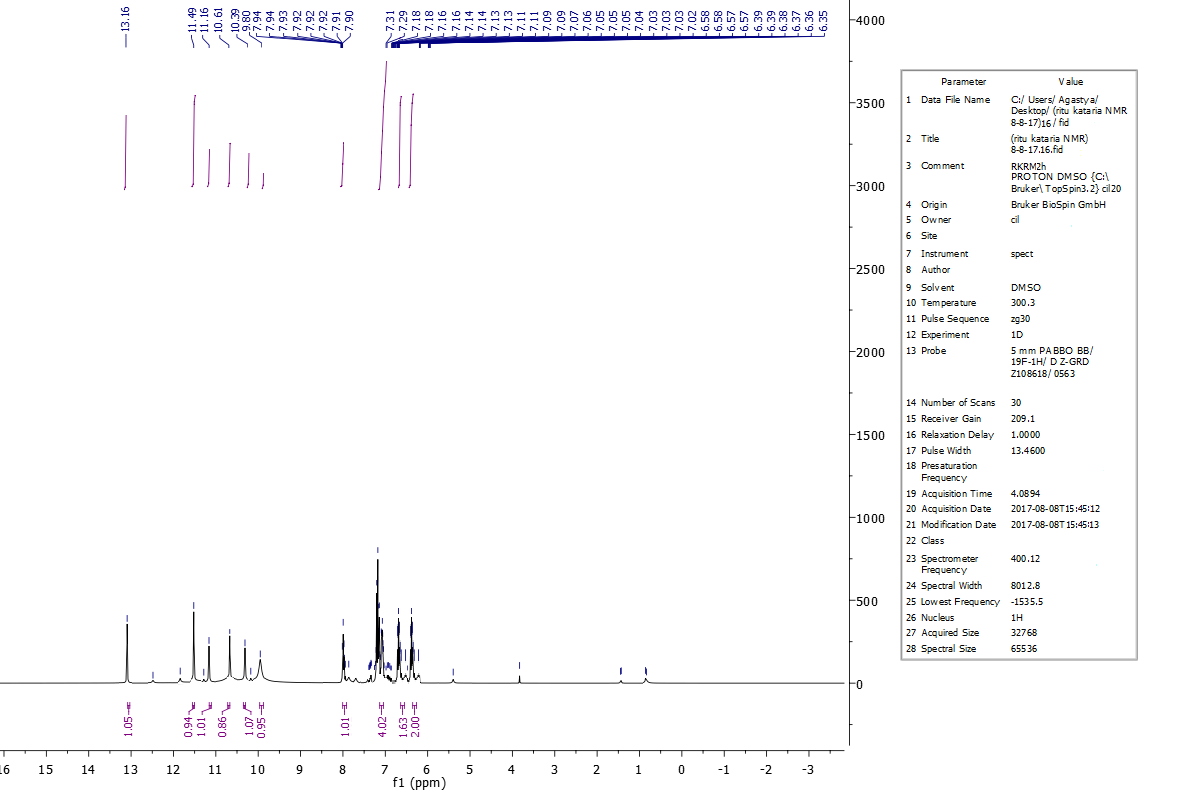


^1^HNMR of Compound M2h


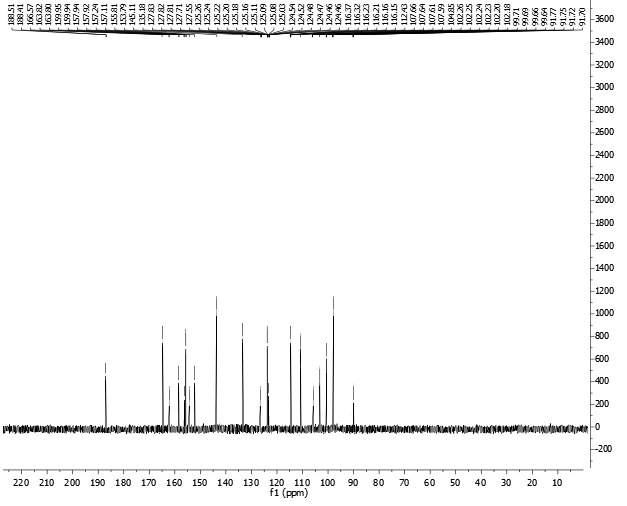


^13^CNMR of Compound M2h


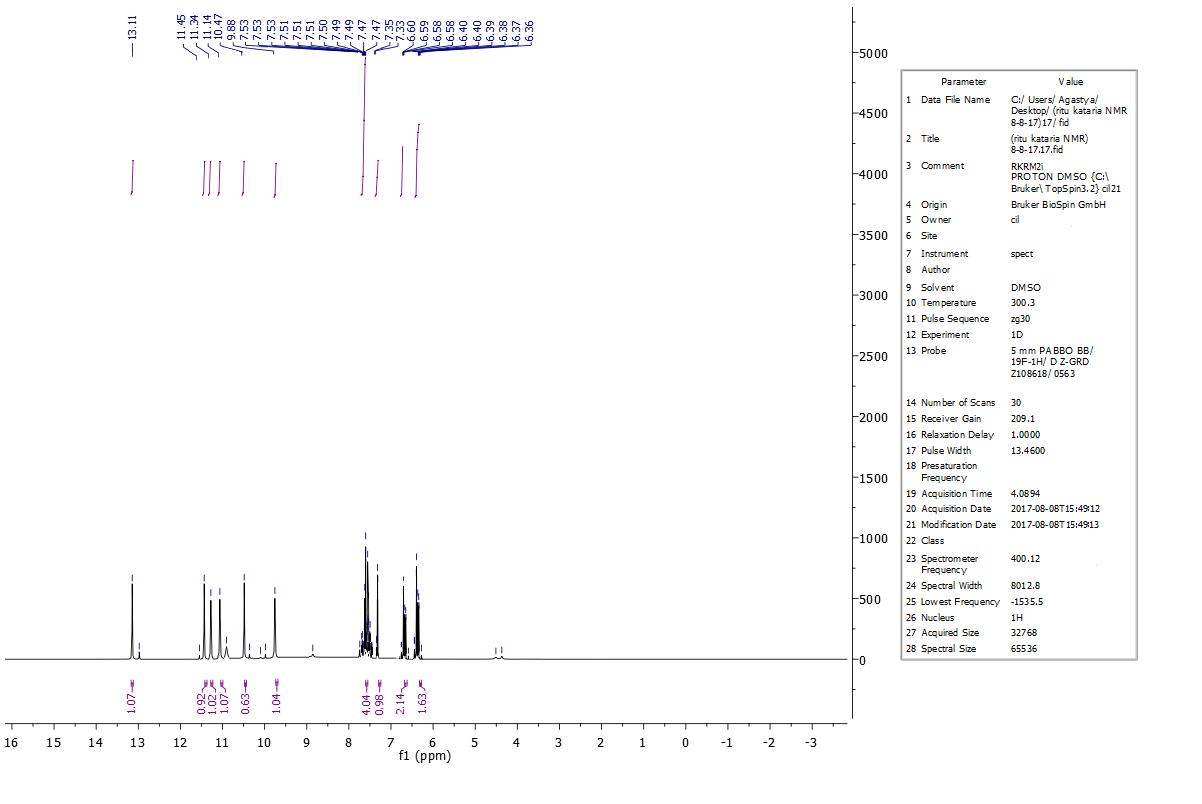


^1^HNMR of Compound M2i


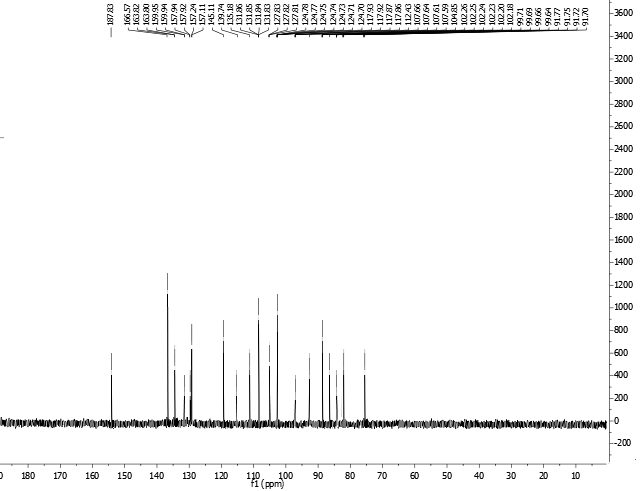


^13^CNMR of Compound M2i


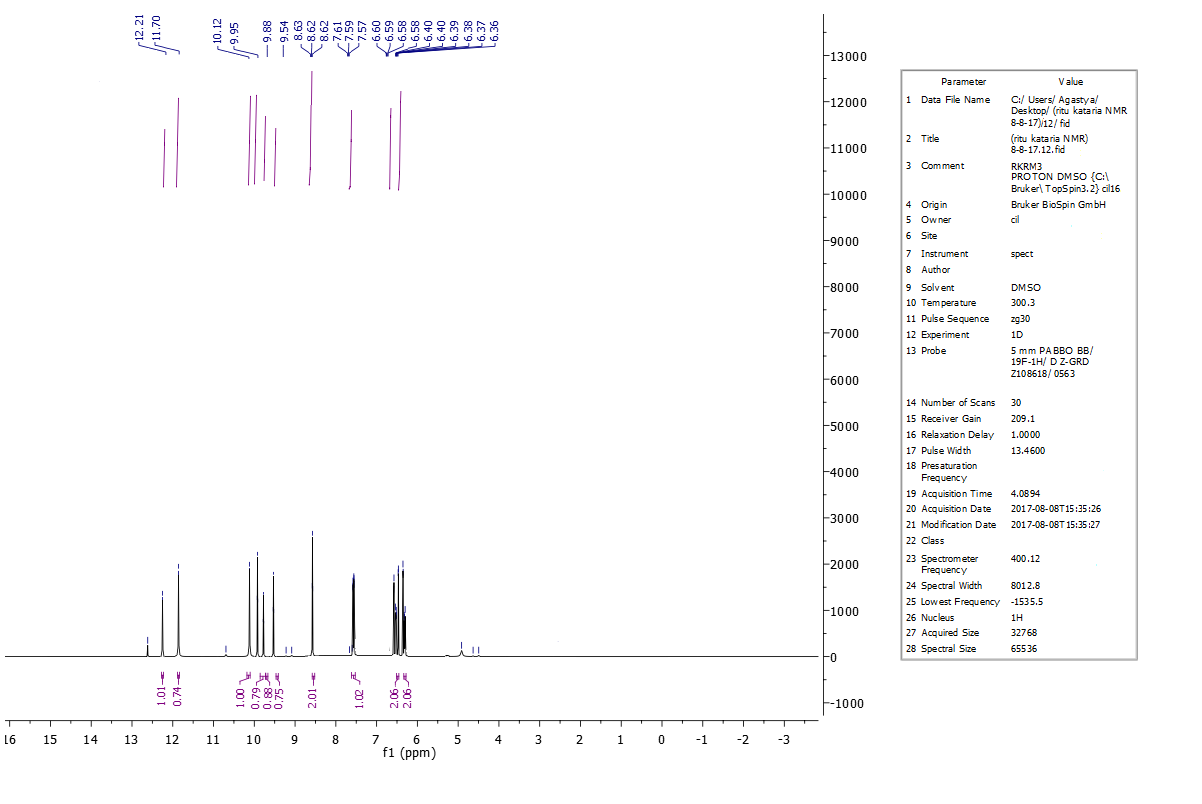


^1^HNMR of Compound M3


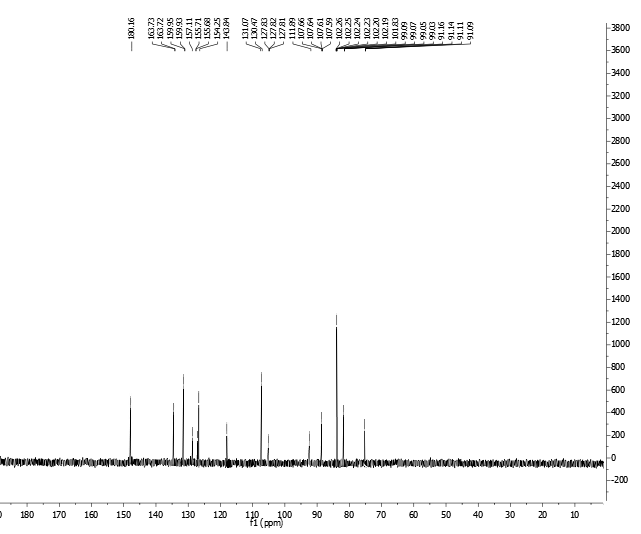


^13^CNMR of Compound M3


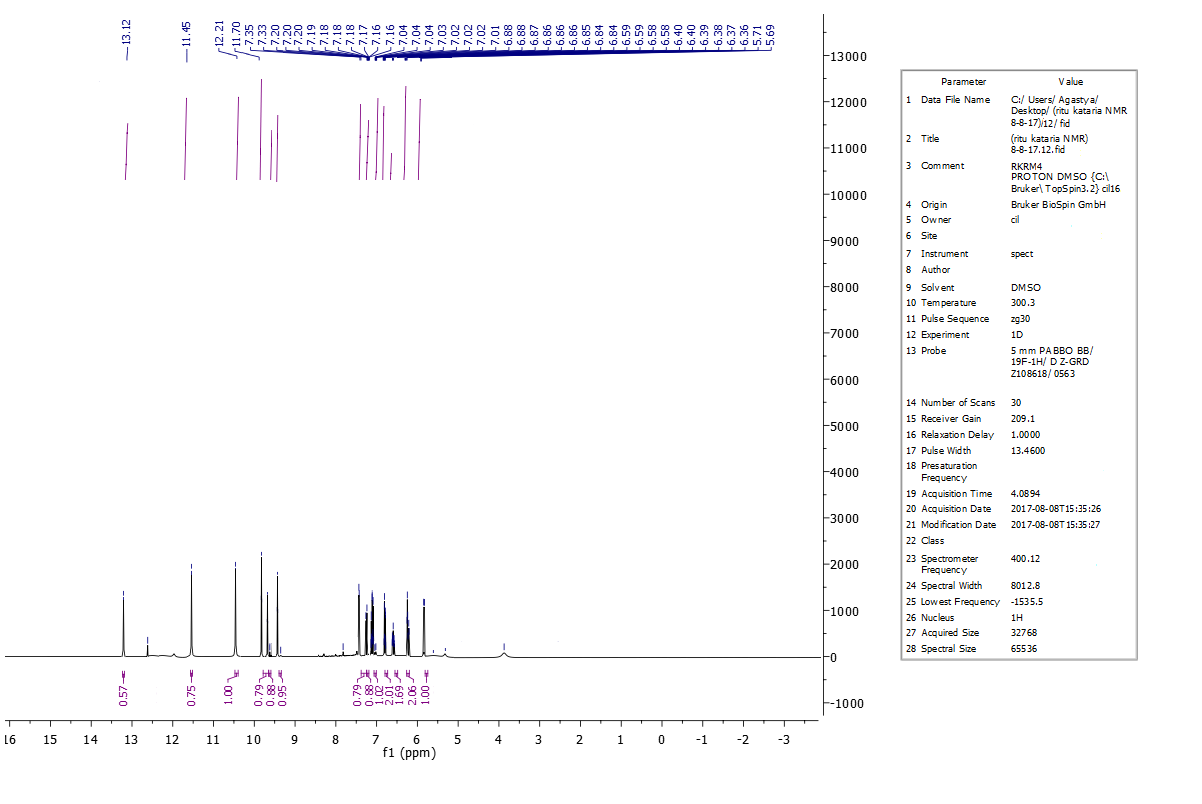


^1^HNMR of Compound M4


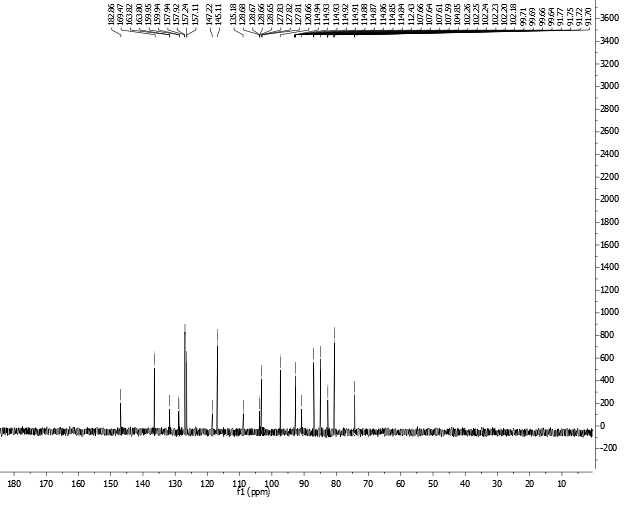


^13^CNMR of Compound M4


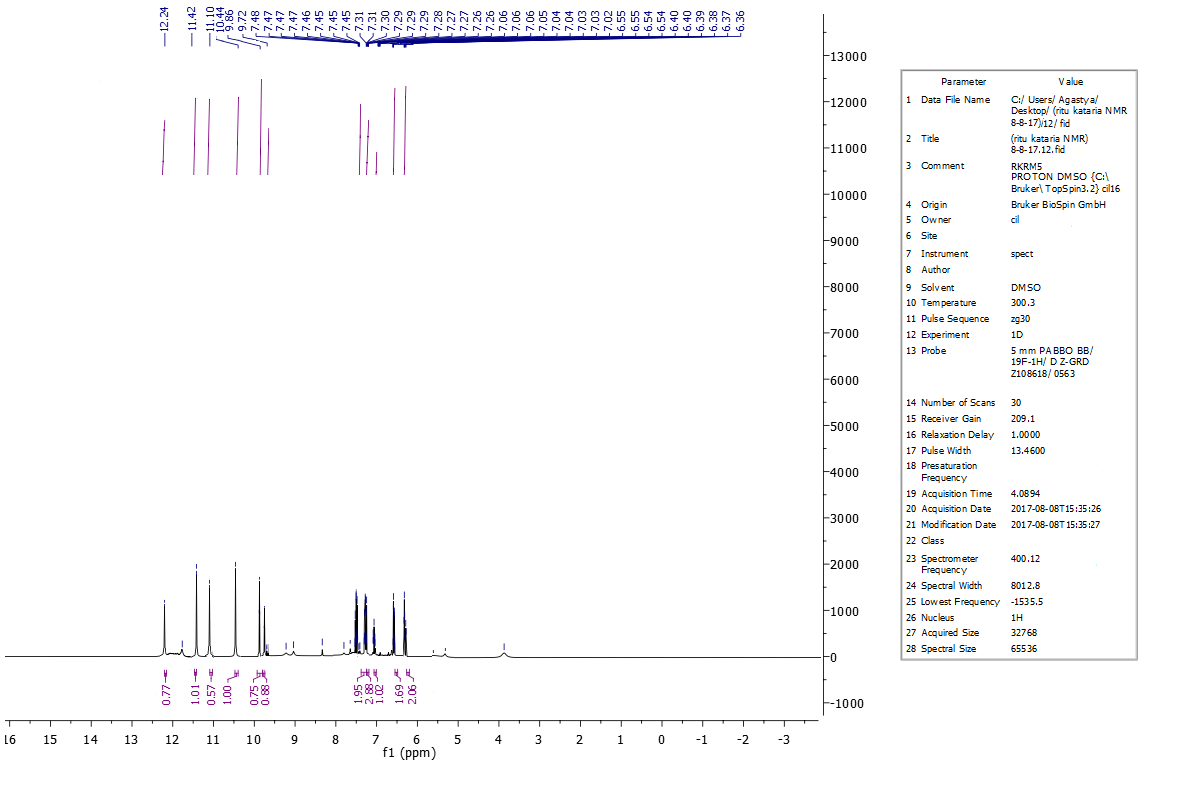


^1^HNMR of Compound M5


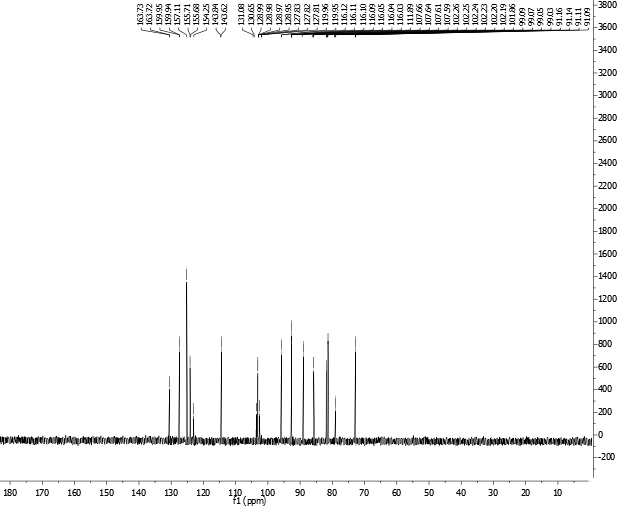


^13^CNMR of Compound M5


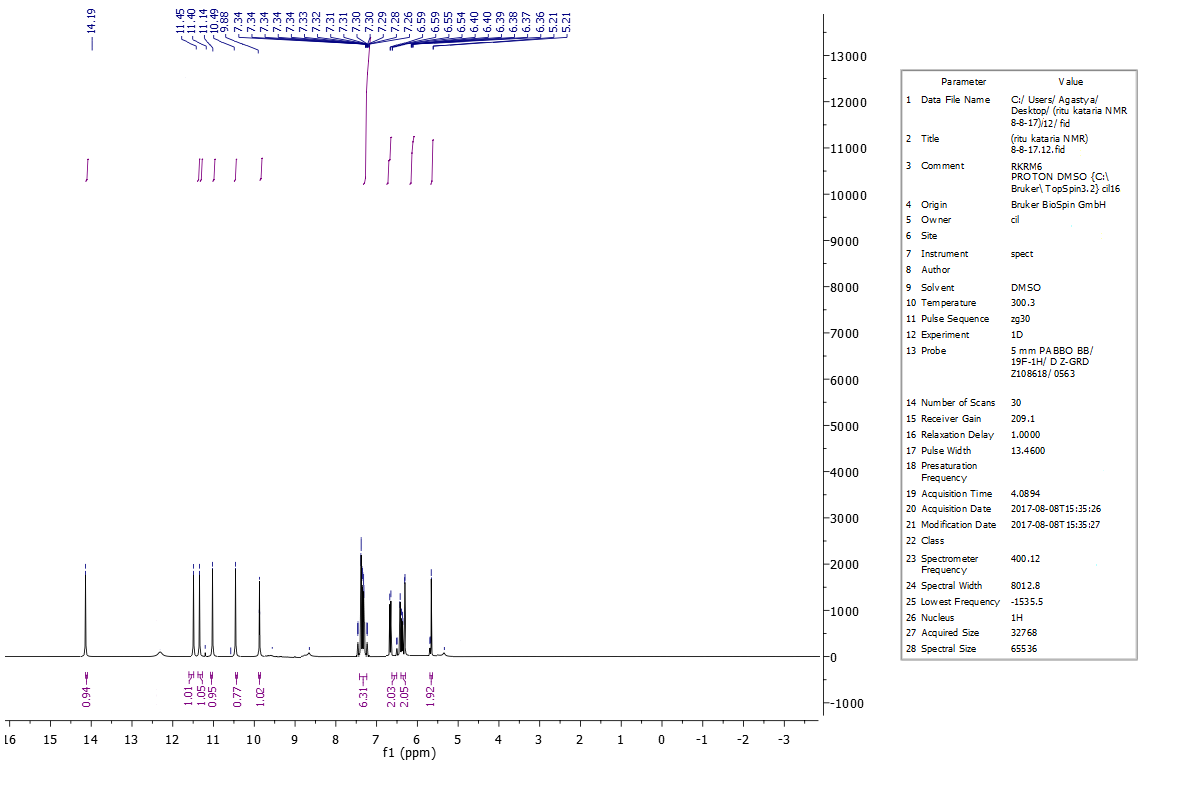


^1^HNMR of Compound M6


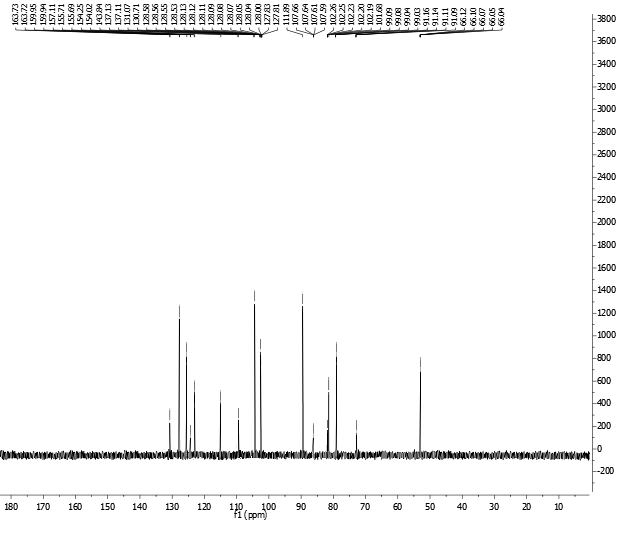


^13^CNMR of Compound M6
